# *BMAL1* Regulates the Daily Timing of Colitis

**DOI:** 10.3389/fcimb.2022.773413

**Published:** 2022-02-09

**Authors:** Zainab Taleb, Vania Carmona-Alcocer, Kyle Stokes, Marta Haireek, Huaqing Wang, Stephen M. Collins, Waliul I. Khan, Phillip Karpowicz

**Affiliations:** ^1^ Department of Biomedical Sciences, University of Windsor, Windsor, ON, Canada; ^2^ Department of Pathology and Molecular Medicine, McMaster University, Hamilton, ON, Canada; ^3^ Farncombe Family Digestive Health Research Institute, McMaster University, Hamilton, ON, Canada

**Keywords:** circadian clock, inflammatory bowel disease, gastrointestinal tract, inflammation, regeneration

## Abstract

Many physiological functions exhibit circadian rhythms: oscillations in biological processes that occur in a 24-hour period. These daily rhythms are maintained through a highly conserved molecular pacemaker known as the circadian clock. Circadian disruption has been proposed to cause increased risk of Inflammatory Bowel Disease (IBD) but the underlying mechanisms remain unclear. Patients with IBD experience chronic inflammation and impaired regeneration of intestinal epithelial cells. Previous animal-based studies have revealed that colitis models of IBD are more severe in mice without a circadian clock but the timing of colitis, and whether its inflammatory and regenerative processes have daily rhythms, remains poorly characterized. We tested circadian disruption using *Bmal1^-/-^* mutant mice that have a non-functional circadian clock and thus no circadian rhythms. Dextran Sulfate Sodium (DSS) was used to induce colitis. The disease activity of colitis was found to exhibit time-dependent variation in *Bmal1^+/+^* control mice but is constant and elevated in *Bmal1^-/-^* mutants, who exhibit poor recovery. Histological analyses indicate worsened colitis severity in *Bmal1^-/-^* mutant colon, and colon infiltration of immune system cells shows a daily rhythm that is lost in the *Bmal1^-/-^* mutant. Similarly, epithelial proliferation in the colon has a daily rhythm in *Bmal1^+/+^* controls but not in *Bmal1^-/-^* mutants. Our results support a critical role of a functional circadian clock in the colon which drives 24-hour rhythms in inflammation and healing, and whose disruption impairs colitis recovery. This indicates that weakening circadian rhythms not only worsens colitis, but delays healing and should be taken into account in the management of IBD. Recognition of this is important in the management of IBD patients required to do shift work.

## Introduction

The circadian clock is a highly conserved endogenous molecular system which drives the oscillation of biological rhythms with a 24-hour period (Takahashi, 2017). These rhythms occur in anticipation of constant environmental changes such as light-dark photoperiod to optimize animal health with the daily changes in its environment. Circadian clock timing is achieved through a transcriptional/translational auto-regulatory feedback loop involving the Clock and Bmal1 positive regulators which transcriptionally activate *Cry1/2* and *Per1/2/3*, their negative regulators (Shearman, 2000). Ror group genes and Nr1d1-2 (also known as Reverbα-β) act as a secondary arm of the clock through the respective activation and repression of *Bmal1* expression (Preitner et al., 2002; Sato et al., 2004). These transcriptional controls allow the clock to oscillate in a 24-hour rhythmic fashion.

Circadian rhythms occur throughout the body and are synchronized in a hierarchical manner (Reppert and Weaver, 2002; Liang et al., 2015). The suprachiasmatic nucleus of the hypothalamus receives photoperiod signals from the retinal ganglion cells of the eye to synchronize its circadian clock to the daily light-dark cycle. This drives rhythms in body temperature and hormones that are thought to synchronize circadian clocks in other tissues. However, the circadian clock is not photoperiod-dependent, which means that it must be resynchronized to environmental cycles to promote health. Shift workers, whose circadian rhythms are disrupted, have been shown to have a higher incidence of Inflammatory Bowel Disease (IBD) (Sonnenberg, 1990). Studies have shown that disruption of the circadian clock by sleep disturbance increase the risk of IBD (Ananthakrishnan et al., 2014) as well as increased disease severity in patients (Keefer et al., 2006; Graff et al., 2011; Swanson et al., 2011).

The mucosal layer of the colon serves as a primary barrier against microbes residing in the gut. This epithelial cell layer forms a series of inward folds known as the crypts of Lieberkühn (Clevers, 2013). A dynamic population of immune cells is interspersed in the epithelium and below in the lamina propria. The cells of the colon epithelium are constantly replenished: the base of the crypt contains intestinal stem cells that divide constantly, and their progeny differentiate into colonocytes/enterocytes and secretory goblet cells and enteroendocrine cells (Marshman et al., 2002). These epithelial cells are joined together by tight junctions and the entire barrier is covered by a mucus layer which reduces direct contact with microbiota and chemicals that might trigger an immune response (Chelakkot et al., 2018). During intestinal regeneration, epithelial and immune cells exhibit cooperation in order to maintain gut homeostasis. Studies have shown that inflammatory cytokines such as IL-6 and IL-22 drive intestinal stem cell regeneration *via* the Jak/Stat and Hippo pathways (Taniguchi et al., 2015). Efficient regeneration of the colon and the maintenance of colonic crypt structures is necessary for resolving tissue injury and preventing microbial infection in order to prevent infectious and inflammatory diseases (Karin and Clevers, 2016).

The circadian clock is present in nearly every cell of the body including the colon (Sládek et al., 2007; Hoogerwerf et al., 2008), where circadian rhythms are entrained by both photoperiod as well as timed feeding (Polidarová et al., 2011). Circadian rhythms are thought to regulate many aspects of gastrointestinal regeneration including the proliferation of mucosal cells (Scheving et al., 1978) and their migration as they differentiate (Qiu et al., 1994). Certain physiological functions of the colon also demonstrate circadian timing, for example electrolyte absorption (Soták et al., 2011) and regulation of barrier function (Summa et al., 2013). Disruption of circadian rhythms through light-dark phase shifts has been shown to drive illness, including metabolic effects that lead to increased obesity (Turek et al., 2005) as well as colitis (Preuss et al., 2008). Immune cells including innate lymphoid type 3 cells, monocytes (Nguyen et al., 2013), macrophages, and natural killer cells (Arjona and Sarkar, 2005) have circadian clock driven rhythms as well. However, the circadian rhythms of colon epithelial cells and immune system cells during colitis remain unclear.

Although the pathology of IBD is an ongoing topic of research, it is generally accepted that mucosal erosion or injury leads to the penetration of microbes or microbial-derived molecules past the epithelium to activate immune cells which induce pro-inflammatory cytokines (Xavier and Podolsky, 2007; Salim and Söderholm, 2011). As a result, patients with IBD experience continuous inflammation along with impaired regeneration of intestinal epithelial cells (Neurath, 2014; Zwarycz et al., 2019). In laboratory mice, DSS is an effective and widely-used model of IBD (Okayasu et al., 1990). Recent studies have shown that disrupting genetic components of the circadian clock mechanism including the genes *Per1/2*, *Nr1d1*, and *Rorα* result in increased colitis severity (Pagel et al., 2017; Wang et al., 2018; Oh et al., 2019). These studies link the loss of different circadian clock components to IBD pathogenesis, suggesting circadian clock genes are important regulators of colitis. However, the daily changes in inflammation and, in particular, daily changes in epithelial regeneration as a result of disrupting the circadian rhythms are not clear. In addition, the loss of *Nr1d1* or *Rorα* do not disrupt circadian rhythms in the body, as these genes are redundant and circadian clock activity persists. We therefore investigated the role of the clock in IBD using *Bmal1^+/+^* (control) and *Bmal1^-/-^* (null mutant) mice, that lack a functional circadian clock. Colitis dampens circadian rhythmicity but does not abolish it, both at the transcriptional and protein level. We observed significantly increased severity of DSS-colitis in *Bmal1^-/-^* mutant mice. Disease activity and inflammatory response in control *Bmal1^+/+^* mice was shown to be time-dependent, revealing that daily rhythms occur during IBD pathogenesis. Our results support the critical role of the circadian clock in colitis and highlight that the clock affects the daily timing of colon epithelial and immune system cell activity.

## Materials and Methods

### Animal Housing and Breeding

The animal studies outlined were reviewed and approved by animal care regulatory boards at McMaster University (#AUPP 19-02-09), or the University of Windsor (#AUPP 20-17). *Bmal1^+/-^* mice (Jackson Laboratories, Bar Harbor, *B6.129-Arntltm1Bra/J* stock #009100) and *Per2::Luciferase* mice (Jackson Laboratories, Bar Harbor, *B6.129S6-Per2tm1Jt/J* stock #006852) were housed in a barrier facility under a 12-hour light/12-hour dark (LD) cycle (with lights on at 7am/Zeitgeber Time 0, and light off at 7pm/Zeitgeber Time 12). Litter mates were used in experiments and were co-housed prior to testing. Normal chow was administered *ad libitum*.

### DSS Acute Colitis Model

**Figure 1:**
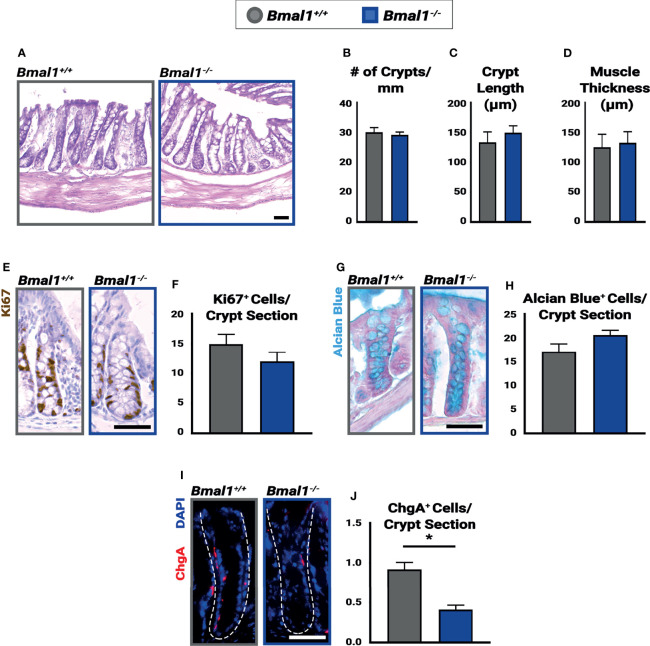
*Bmal1^-/-^* mutants have no significant colon phenotype but show decreased numbers of enteroendocrine cells. Untreated *Bmal1^+/+^* control and *Bmal1^-/-^* mutant colon tissues were collected at ZT4 and examined for epithelial differences. **(A)** Hematoxylin-eosin staining shows no difference in morphology between genotypes. No difference was seen in **(B)** number of crypts along the colon (crypts/mm) (Unpaired *t*-test, p=0.6627), **(C)** average crypt length (µm) (Unpaired *t*-test, p=0.4786), **(D)** or average muscle thickness (µm) (Unpaired *t*-test, p=0.8027). **(E, F)** Goblet cells (marked by Alcian Blue) are present in similar amounts in *Bmal1^+/+^* control and *Bmal1^-/-^* mutant colons (Unpaired *t*-test, p=0.2334). **(G, H)** *Bmal1^+/+^* control and *Bmal1^-/-^* mutant crypts were tested at three timepoints over the day (ZT0, ZT4, and ZT12) in order to quantify Ki67+ (brown; labels cells in S/G2/M phases) proliferating cells. No significant differences in cell proliferation were detected at any time (graph combines all three times, Unpaired *t*-test, p=0.1427). **(I, J)** A decrease in enteroendocrine cell population (Chromogranin A in red) is noted in *Bmal1^-/-^* mutant colons (Unpaired *t*-test, *p=0.0012). (All error bars represent SEM. Scale bar = 50µm. Dashed lines indicate crypt.).

**Figure 2:**
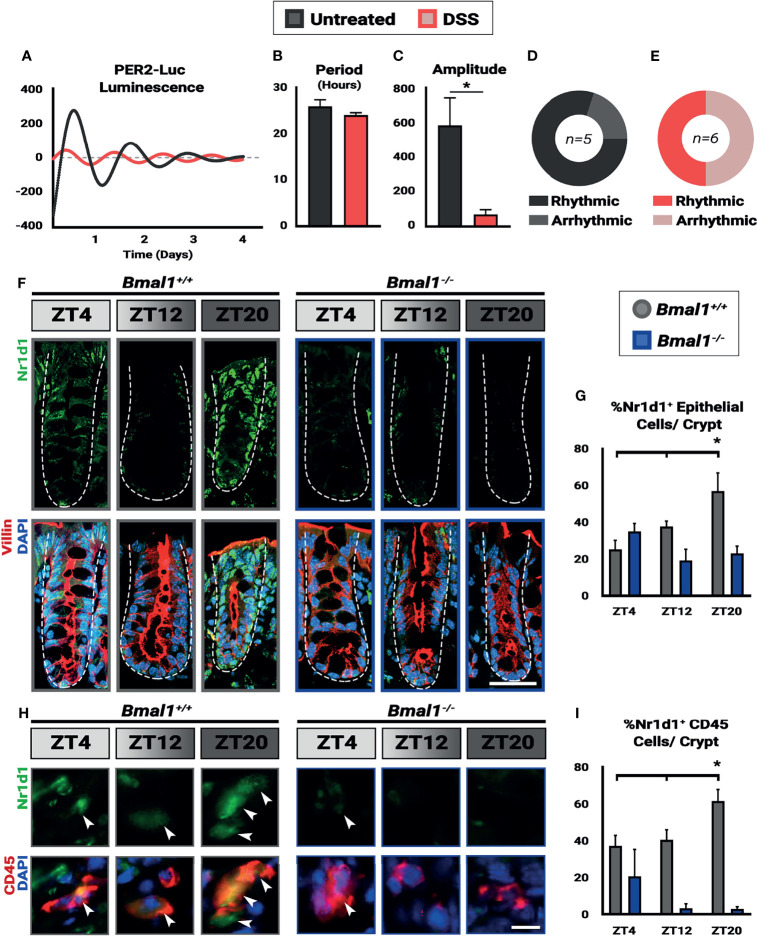
Colitis dampens circadian rhythms in the colon. Mice were treated with 4% DSS and collected at Zeitgeber times (ZT) 4, 12, and 20. **(A)** Colon explants show dampened circadian rhythms in *Per2::Luc* animals treated with DSS. **(B)** Despite the lower amplitude of the rhythms in DSS-treated mice, no difference in period is present in the colon tissue (Unpaired *t*-test, p=0.3131). **(C)** Amplitude in *Per2::Luc* rhythms is decreased in DSS-treated explants (Unpaired *t*-test, *p=0.0410). **(D)** 80% of untreated explants exhibit high-amplitude rhythms. **(E)** 50% of DSS treated explants exhibit low amplitude rhythms, and the rest are arrhythmic. **(F)** Nr1d1 protein (in green) is expressed in *Bmal1* ^+/+^ control Villin^+^ epithelial cells (in red) (Scale bar = 50µm. Dashed lines indicate crypt outline). **(G)** The peak in Nr1d1 expression in Villin^+^ epithelial cells occurs at ZT20, and Nr1d1 protein is present at decreased levels in *Bmal1* ^-/-^ mutant tissue (*Bmal1^+/+^* One-way ANOVA, *p=0.0383, F=5.898; Tukey’s test, ZT20 *p=0.0134). **(H)** Nr1d1 protein (in green) is expressed in *Bmal1* ^+/+^ control colon lamina propria CD45+ blood cells (in red) (Arrowheads indicate examples of CD45^+^ cells. Scale bar = 10µm). **(I)** The peak in Nr1d1 expression in CD45+ cells also occurs at ZT20, and Nr1d1 protein is present at decreased levels in *Bmal1* ^-/-^ mutants (*Bmal1^+/+^* One-way ANOVA, *p=0.0483. F=5.238; Tukey’s test, ZT12 *p=0.0391, ZT20 *p=0.0011). (All error bars represent SEM.).

**Figure 3:**
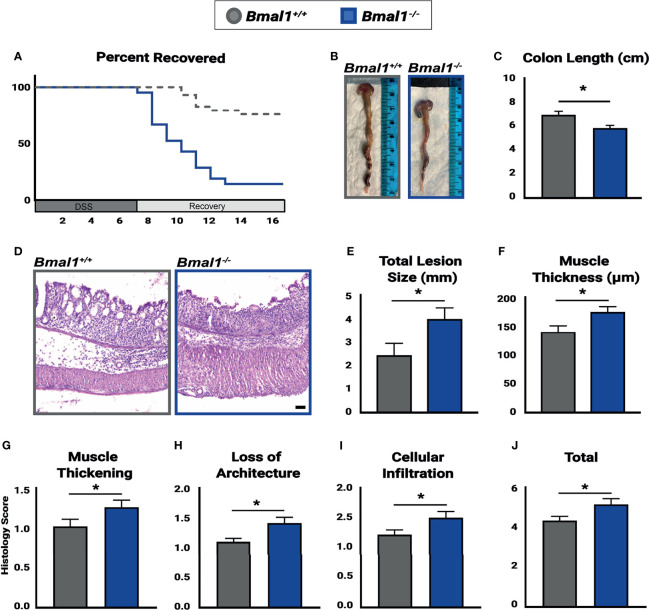
*Bmal1^-/-^* mutant mice have more severe colitis and increased loss of epithelial structure. Mice were treated with DSS, disease activity was monitored daily, and histopathology was determined. Colon tissues were collected at the end of DSS treatment across the full 24 hours (ZT0, ZT4, ZT8, ZT12, ZT18 & ZT20). **(A)** *Bmal1^-/-^* mutant mice do not recover from colitis compared to *Bmal1^+/+^* controls. Kaplan-Meier curve show mutants reach study endpoint at an increased rate (Mantel-Cox test, *p<0.0001). **(B)** Representative images of colons. **(C)** *Bmal1^-/-^* mutant mice exhibit decreased colon length. (Unpaired *t*-test, *p=0.0247). **(D)** Representative images of Hematoxylin-Eosin staining in control *versus Bmal1^-/-^* mutants. **(E)** Total lesion size (area of tissue section completely lacking crypts and normal colon morphology) size throughout the colon was measured in Swiss rolls and averaged per animal. *Bmal1^-/-^* mutant mice exhibit increased size of lesioned/amorphous regions compared to controls. (Unpaired *t*-test, *p=0.0298). **(F)** Increased muscle thickness is also seen in *Bmal1* ^-/-^ mice, indicating more severe inflammation (Unpaired *t*-test, *p=0.0201). **(G–J)** Histology scores were assessed based on the severity of colitis in tissue sections. *Bmal1^-/-^* mutant tissue contains: **(G)** Increased muscle thickening (Unpaired *t*-test, *p= 0.0405); **(H)** Increased loss of crypt architecture (Unpaired *t*-test, *p=0.0072); **(I)** Increased cellular infiltration (Unpaired *t*-test, *p=0.0339). These factors contribute to **(J)** increased total histology scores in the *Bmal1^-/-^* mutant colon with colitis (Unpaired *t*-test, *p=0.0234). (All error bars represent SEM. Scale bar = 50µm).

**Figure 4:**
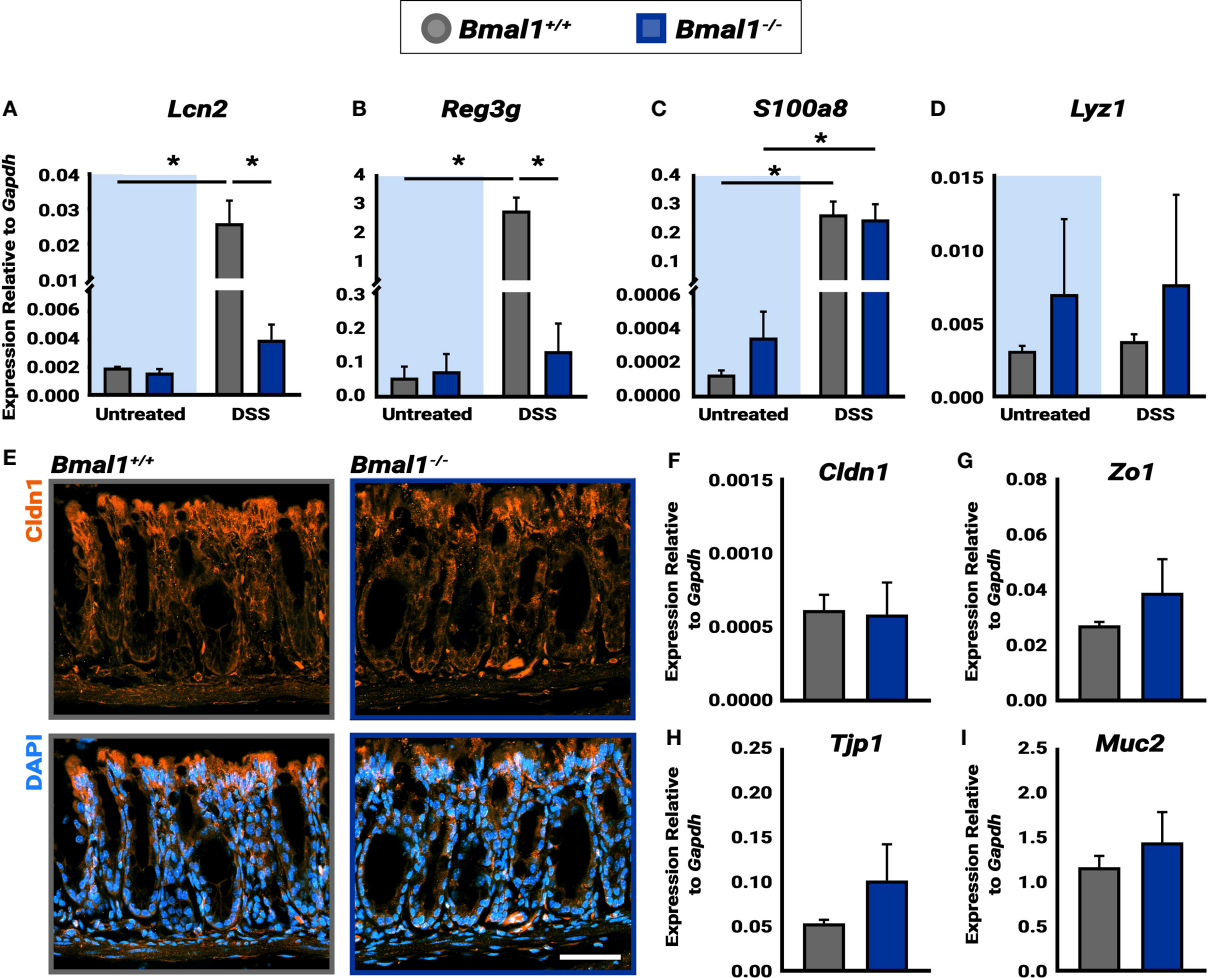
*Bmal1^-/-^* mutant mice exhibit increased AMP production during DSS-colitis despite similar epithelial barrier integrity. **(A)** Increased *Lcn2* expression in DSS-colitis treated *Bmal1^+/+^* colon tissue lysate collected at ZT16. (Tukey’s test, DSS *p=0.0150, Untreated vs. DSS *Bmal1^+/+^* *p=0.0147). **(B)** Increased *Reg3g* expression in DSS-colitis treated *Bmal1^+/+^* colon tissue lysate collected at ZT16. (Tukey’s test, DSS *p=0.0003, Untreated vs. DSS *Bmal1^+/+^* *p=0.0005). **(C)** Increased *S100a8* expression in *Bmal1* ^+/+^ control and *Bmal1^-/-^* mutant colon tissue following DSS-colitis. (Tukey’s test, *Bmal1* ^+/+^ untreated *vs.* DSS *p=0.0085, *Bmal1^-/-^* untreated *vs.* DSS *p=0.0270). **(D)** No difference in *Lyz1* expression in *Bmal1* ^+/+^ control and *Bmal1^-/-^* mutant colon in both untreated and DSS-colitis conditions. (Tukey’s test, ns). **(E)** Representative images of the tight junction protein Cldn1 (in orange) in *Bmal1* ^+/+^ control and *Bmal1^-/-^* mutant colon tissue treated with DSS-colitis. No clear changes were observed in Cldn1 by antibody. In addition, no significant differences in transcriptional expression were detected in **(F)** *Cldn1*, **(G)** *Zo1*, **(H)** *Tjp1*, and **(I)** *Muc1* genes. (Unpaired *t-*test, *Cldn1* p=0.8932, *Zo1* p=0.3116, *Tjp1* p=0.2236, *Muc2* p=0.4295). (Gene expression normalized to *Gapdh.* All error bars represent SEM. Scale bar = 50µm).

**Figure 5:**
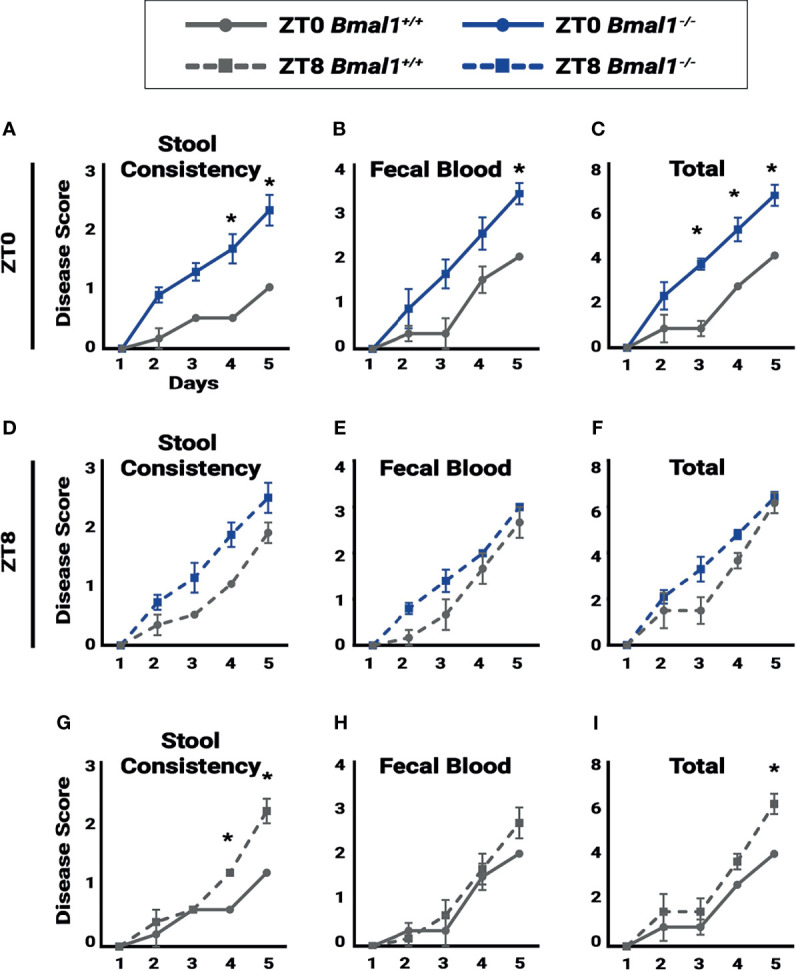
Colitis is time dependent. Disease score was assessed for individual mice to test for the severity of symptoms associated with colitis during a 5 day, 5% DSS treatment. A lower disease score is exhibited in *Bmal1^+/+^* control mice at ZT0 due to: **(A)** decreased stool consistency (Two-way ANOVA, *p=0.0042, F_(4,25)_=5.004; Tukey’s test, Day 4 *p=0.0009, Day 5 *p=0.0002) and **(B)** decreased fecal blood on the final day of treatment and (Two-way ANOVA, p=0.1199, F_(4,25)_=2.036; Tukey’s test, Day 5 *p=0.0496). **(C)** Overall total disease score (Two-way ANOVA, *p=0.0093, F_(4,25)_=4.241; Tukey’s test, Day 3 *p= 0.0016, Day 4 *p=0.0067, Day 5 *p=0.0033). No difference between *Bmal1^+/+^* control and *Bmal1^-/-^* mutant mice was seen in **(D)** stool consistency, **(E)** fecal blood, and **(F)** overall total disease score at ZT8 upon induction of DSS-colitis (Two-way ANOVA, ns; Tukey’s test, ns). Time dependence in disease severity is apparent in circadian-competent mice when comparing *Bmal1^+/+^* controls at ZT0 and ZT8. **(G)** Increased disease activity in stool consistency at ZT8 (Two-way ANOVA, *p=0.0006, F_(4,20)_=7.883; Tukey’s test, Day 4 *p=0.0252, Day 5 *p<0.0001). **(H)** Although no time dependence is seen in fecal blood (Two-way ANOVA, p=0.4930, F_(4,20)_=0.8810; Tukey’s test, ns), **(I)** Increased total disease activity upon induction of DSS at ZT8 is observed (Two-way ANOVA, p=0.1610, F_(4,20)_=1.839; Tukey’s test, Day5 *p=0.0367). (All error bars represent SEM).

**Figure 6:**
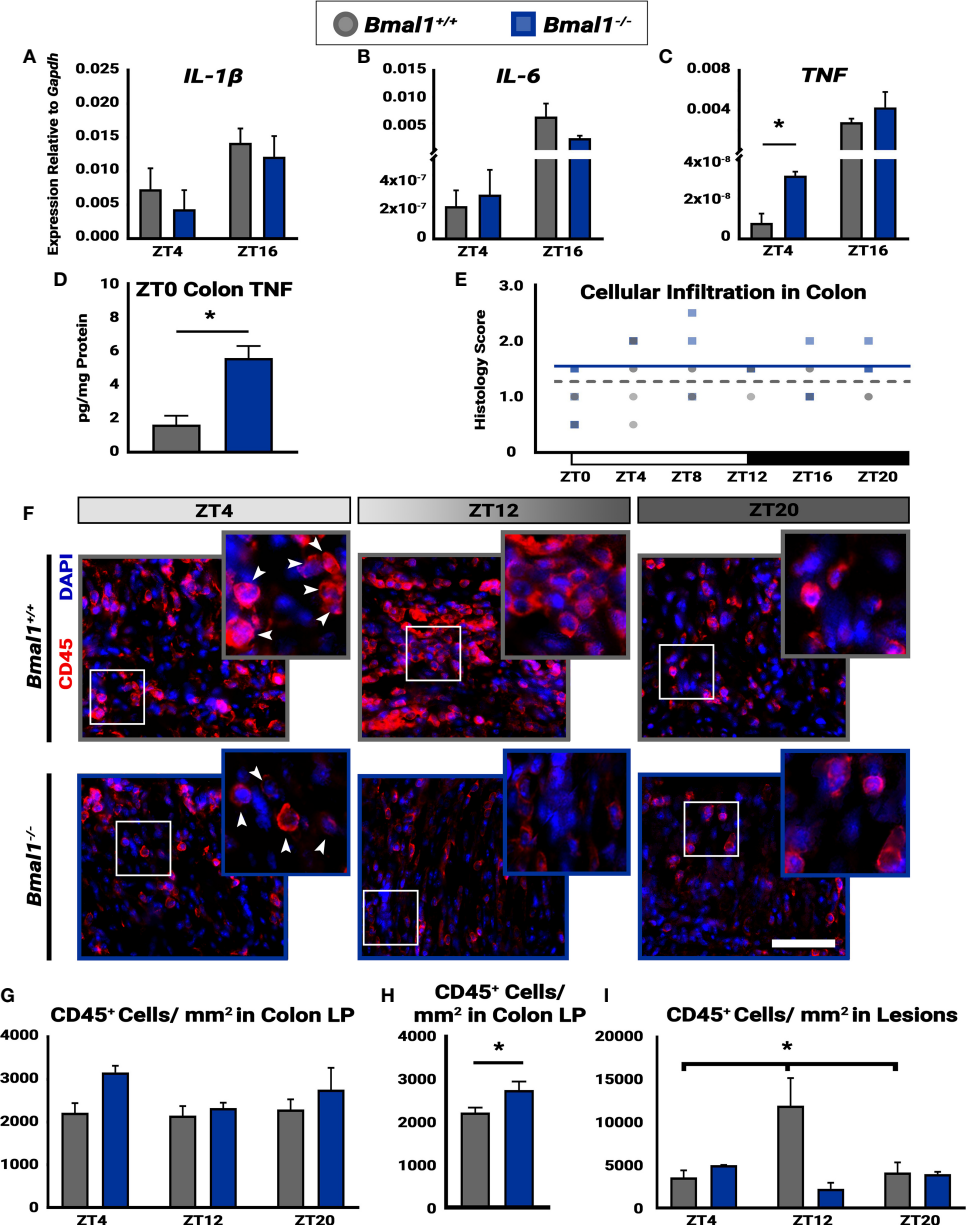
Time-dependent cellular infiltration is disrupted in *Bmal1^-/-^* mutant mice. Mice were given DSS treatment to induce colitis, and samples were collected at the indicated times. No changes in expression of **(A)** IL-1β (Unpaired *t*-test, ZT4 p=0.5191, ZT16 p=0.4409) or **(B)** IL-6 between *Bmal1* ^+/+^ control and *Bmal1^-/-^* mutant mice at ZT4 and ZT16 (Unpaired *t*-test, ZT4 p=0.7145, ZT16 p=0.2605). **(C)** Increased *TNF* expression is present in *Bmal1^-/-^* colon tissue at ZT4, but no difference is present at ZT16. (Unpaired *t*-test, ZT4 *p=0.0100, ZT16 p=0.2719). **(D)** Increased TNF protein concentration in whole colon tissue lysate of *Bmal1^-/-^* mutants is evident. (Unpaired *t*-test, *p=0.0115). **(E)** The histology scores of the whole colon indicate no variation in cellular infiltration across 24 hours (One-way ANOVA, *Bmal1* ^+/+^ p= 0.2388, F= 1.493; *Bmal1^-/-^* p=0.1274, F= 2.039). **(F)** Images of infiltrating blood cells into colon lesions are marked by CD45 transmembrane protein, in red. (Arrowheads in closeup indicate examples of CD45^+^ cells. Scale bar = 50µm). **(G)** Infiltrating blood (CD45^+^) cells in the lamina propria (LP) from normal regions of the colon exhibit no time-dependent changes in either *Bmal1^+/+^* or *Bmal1^-/-^* mice. (One-way ANOVA, *Bmal1* ^+/+^ p= 0.9109, F= 0.0948; *Bmal1^-/-^* p=0.2548, F= 1.732) **(H)** The average of all CD45^+^ cellular infiltration across time points (ZT4, 8, and 20) indicates an increase in *Bmal1^-/-^* mutant mice is present (Unpaired *t*-test, *p=0.0360). **(I)** In the colon lesions, however, infiltrating CD45^+^ blood cells exhibit time-dependent changes in *Bmal1^+/+^* control mice, with an increase at ZT12, that is absent in the *Bmal1^-/-^* mutant. (*Bmal1^+/+^* One-way ANOVA, *p= 0.0474, F=5.287; Tukey’s test, *Bmal1^+/+^* - *Bmal1^-/-^* ZT12, *p=0.0056). (Gene expression normalized to *Gapdh*. All error bars represent SEM).

**Figure 7:**
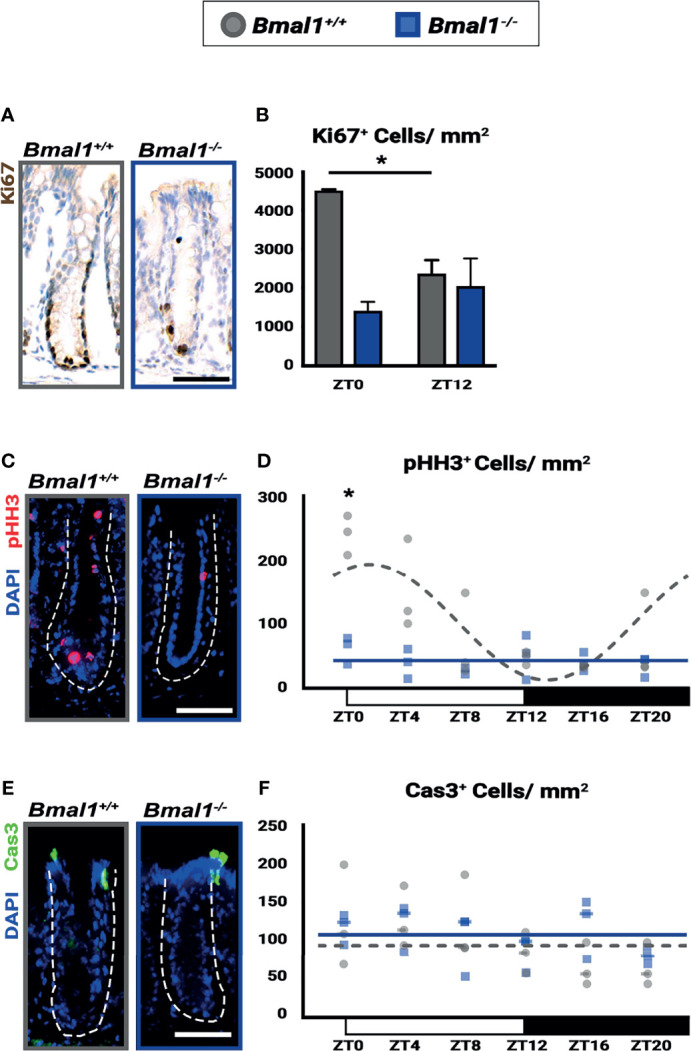
Diurnal rhythms in regeneration are disrupted and decreased in *Bmal1^-/-^* mutant mice. *Bmal1* ^+/+^ control and *Bmal1^-/-^* mutant mice were collected after DSS treatment to induce colitis, and morphologically normal crypts were examined. **(A, B)** Proliferation (Ki67 shown in brown) is increased in *Bmal1* ^+/+^ control compared to the *Bmal1^-/-^* mutant at ZT0, and is also significantly increased at ZT0 compared to ZT12 in the *Bmal1* ^+/+^ control (Two-way ANOVA, *p=0.0096, F_(1,8)_=11.43; Tukey’s test, ZT0 *Bmal1^+/+^* - *Bmal1^-/-^* *p=0.0031, ZT0, 12 *Bmal1^+/+^* *p=0.0253). In contrast, proliferation is decreased and does not significantly change between ZT0 and ZT12 in *Bmal1* mutants. **(C, D)** Mitosis (marked by phosphorylated histone-H3, pHH3 in red) is increased and rhythmic in *Bmal1* ^+/+^ control mice with colitis while decreased and arrhythmic in *Bmal1^-/-^* mutants (Two-way ANOVA, *p=0.0021, F_(5,24)_=5.264; Tukey’s test, ZT0 *Bmal1^+/+^* - *Bmal1^-/-^* *p=0.0003; Chronos-Fit, *Bmal1^+/+^* *p=0.0011). **(E, F)** Apoptosis (marked by cleaved Caspase-3, Cas3 in green) does not exhibit significant differences between either *Bmal1* ^+/+^ control and *Bmal1^-/-^* mutant colons and is not expressed rhythmically (Two-way ANOVA, p=0.5329, F_(5, 22)_=0.8444). (All error bars represent SEM. Dashed lines indicate crypt. Scale bar= 50µm).

**Figure 8:**
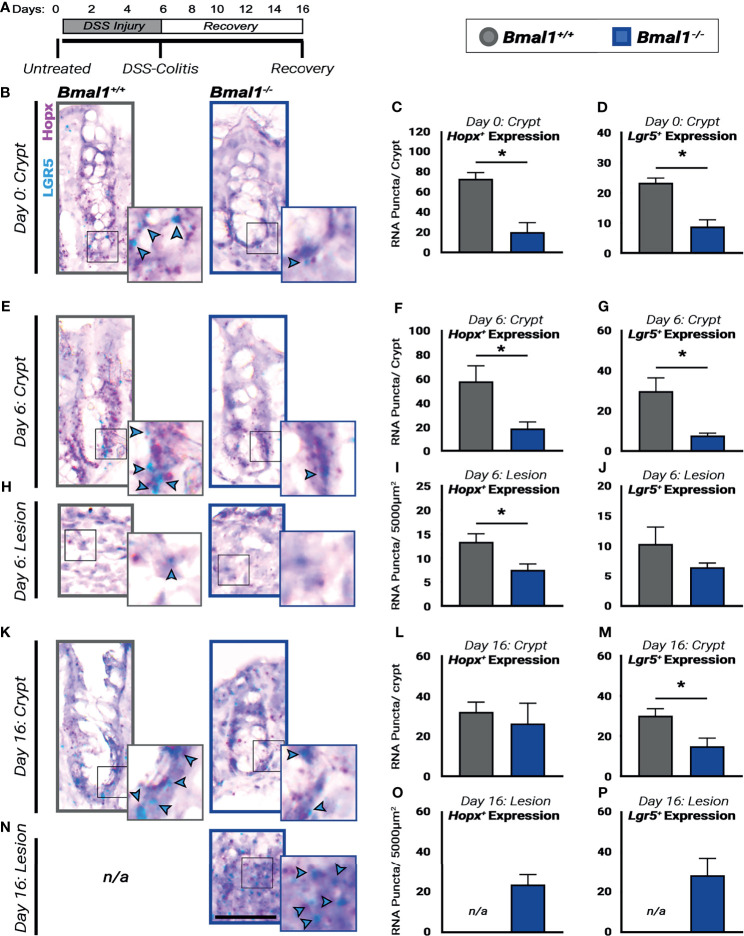
Regenerative pathways are disrupted and decreased in *Bmal1^-/-^* mutant mice. **(A)** Experiment schematic: *Bmal1* ^+/+^ control and *Bmal1^-/-^* mutant mice were collected before DSS treatment (Day 0), after 6 days of 4% DSS treatment to induce colitis (Day 6) and following 10 days of recovery after DSS removal (Day 16). **(B)** Representative images of sections probed for transcriptional expression of the fetal-like regenerative precursor marker *Hopx* (in pink), and the ISC marker *Lgr5* (in blue, highlighted with blue arrow heads) in untreated (Day 0) *Bmal1* ^+/+^ control and *Bmal1^-/-^* mutant mice. *Bmal1^-/-^* mutants show decreased levels of both **(C)** *Hopx* and **(D)** *Lgr5* markers in untreated mice. (Unpaired *t-*test, *Hopx* *p= 0.0091, *Lgr5* *p= 0.0062). **(E)** Representative images of crypts probed for transcriptional expression of *Hopx*, and *Lgr5* in DSS-colitis treated (Day 6) *Bmal1* ^+/+^ control and *Bmal1^-/-^* mutant mice. **(F)** Upon colitis induction at Day 6, *Bmal1^-/-^* mutant mice exhibit a decrease in *Hopx* in crypts. (Unpaired *t-*test, *Hopx* *p=0.0491). **(G)** *Bmal1^-/-^* mutant mice exhibit a decrease in *Lgr5* in crypts. (Unpaired *t-*test, *Lgr5* *p=0.0301). **(H)** Representative images of lesions probed for transcriptional expression of *Hopx*, and *Lgr5* in DSS-colitis treated (Day 6) *Bmal1* ^+/+^ control and *Bmal1^-/-^* mutant mice. **(I)** Lesions of *Bmal1* ^+/+^ control mice also show decreased *Hopx* expression. (Unpaired *t-*test, *Hopx* *p=0.0489). **(J)** Lesions of *Bmal1* ^+/+^ control and *Bmal1^-/-^* mutant mice show low but similar *Lgr5* expression. (Unpaired *t-*test, p=0.2521). **(K)** Representative images of crypts probed for transcriptional expression of *Hopx*, and *Lgr5* following 10 days of recovery (Day 16) *Bmal1* ^+/+^ control and *Bmal1^-/-^* mutant mice. **(L)** After 10 days of recovery, *Bmal1^-/-^* mutant mice exhibit similar *Hopx* expression in crypts. (Unpaired *t-*test, p=0.66226). **(M)** *Bmal1^-/-^* mutant mice exhibit a decrease in *Lgr5* in crypts. (Unpaired *t-*test, *p= 0.0468).**(N)** Representative images of *Bmal1^-/-^* mutant lesion sections probed for transcriptional expression of *Hopx*, and *Lgr5* following 10 days of recovery (Day 16). Sections labeled not/applicable (n/a) indicate that lesions were not present in *Bmal1* ^+/+^ control mice. **(O)** Quantification of *Hopx* expression in *Bmal1^-/-^* mutant lesions following 10 days recovery. **(P)** Quantification of *Lgr5* expression in *Bmal1^-/-^* mutant lesions following 10 days recovery. (All error bars represent SEM. Scale bar = 50µm).

**Figure 9:**
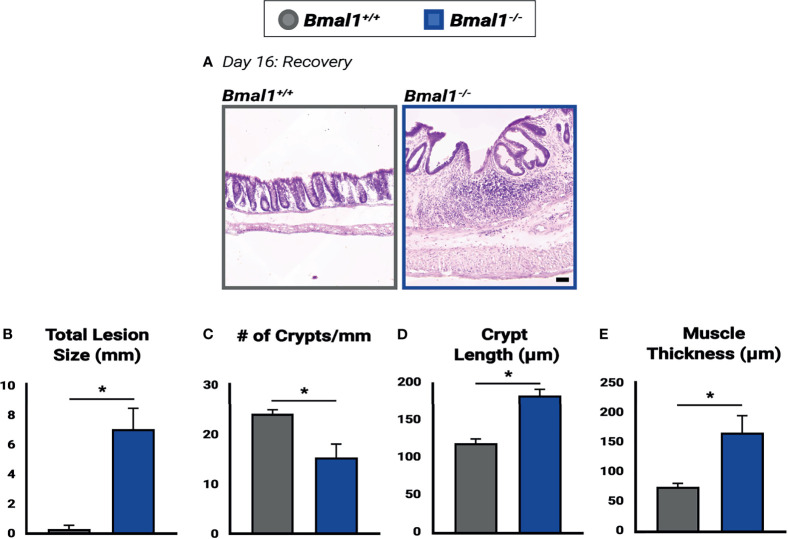
Impaired recovery of *Bmal1^-/-^* mutant colon tissue following DSS-colitis. **(A)** Representative images of Hematoxylin-Eosin staining in control *versus Bmal1^-/-^* mutants. Day 16: Recovered colon tissue was morphologically assessed for resolution of colitis. **(B)** Increased total lesion size is evident in *Bmal1^-/-^* mutant tissue (Unpaired *t*-test, *p=0.0090). **(C)** Decreased crypt density (Unpaired *t*-test, *p=0.0344) is present in *Bmal1^-/-^* mutants, and *Bmal1^-/-^* mutant colons also exhibit **(D)** increased crypt elongation (Unpaired *t*-test, *p=0.0032), and **(E)** increased muscle thickening (Unpaired *t*-test, *p=0.0354) indicating a disrupted regenerative response. (All error bars represent SEM).

Mice aged 12-14 weeks were housed individually for 1 week before beginning the experiments to acclimate to the new cage. Bedding taken from their previous co-housed cages was sprinkled into the new individual cages in order to further control for microbiome effects. Untreated mice were collected following the same 1 week acclimation period (**Figures 1**, **4**, **8**). A 4% (w/v) solution of DSS (MW: 36-50 KDa, MP Biomedicals, Solon, Ohio) dissolved in normal drinking water was administered *ad libitum* to the mice over the course of 6 days (**Figures 2**–**4**, **6**–**9**). Water consumption of normal drinking water and DSS water was measured twice a day (ZT0 and ZT12) and no differences were observed between controls and mutants in the amount of DSS-water consumed (data not shown). This same colitis assay was repeated in McMaster University using a 5% (w/v) solution of DSS in normal drinking water over 5 days (data shown in **Figure 5**).

### Recovery Assay

After the 6-day DSS treatment, the DSS solution was removed and replaced with normal drinking water. Mice were tested 10 days after DSS removal (**Figures 3**, **8**, **9**). Weight and disease activity (see below) was monitored daily. If weight loss exceeded 25% over the duration of 3 days, or the mice appeared lethargic, it was taken as a sign of extreme sickness and the experiment was terminated and the mice humanely euthanized (data is shown in **Figure 3A**).

### Tissue Collection

Mice were euthanized using CO_2_ followed by cervical dislocation at various intervals over 24 hours (Zetigeber Times: ZT0, ZT4, ZT8, ZT12, ZT16, ZT20 as indicated in text). Colon tissue was removed and flushed with ice cold phosphate buffer saline (PBS). The tissue was cut longitudinally to open the lumen, and was then rolled up from the proximal to distal end. The dissected colon tissue was fixed in 4% paraformaldehyde (PFA) (Electron Microscopy Sciences, Hatfield, PA) diluted in PBS for 2 hours. Segments of the proximal, medial and distal colon were collected for gene expression analysis and placed in RNAlater buffer (Qiagen, Germantown, MD) at 4°C overnight (O/N), then stored at -80°C.

### Disease Activity Index

Disease Activity Index (DAI) score was monitored during DSS treatment to evaluate the progression of colitis symptoms. DAI score consisted of daily measurements of weight loss compared to initial weight, stool consistency and bleeding. Each disease criteria is assigned a score based on the severity of the symptom (weight loss 0:<1%, 1: 1–5%, 2: 5–10%, 3: 11-14%, 4:>15%, stool consistency 0: normal, 2: loose stools, 4: diarrhea, stool blood 0: negative, 2: positive, rectal bleeding 4: if present) (Kim et al., 2012).

### Staining and Histology

Fixed colon tissues were washed 3 times in PBS to remove any PFA before being submerged in 30% (w/v) sucrose in PBS for 24 hours at 4°C. The tissue was transferred into Tissue-Tek O.C.T compound (Sakura, Flemingweg, Netherlands) and left at 4°C for 1 hour before being frozen at -80°C. The frozen tissue was sectioned at 10μm using a Leica CM 1950 Cryostat (Leica Biosystems). Sectioned tissues were washed 3 times in room temperature PBS for 5 minutes each. This was followed by antigen retrieval and another 3 washes of PBS for 5 minutes as needed. Tissue morphology was assessed using Hematoxylin & Eosin (Electron Microscopy Sciences, Hatfield, PA), and goblet cells were stained using Alcian Blue (Electron Microscopy Sciences, Hatfield, PA). H&E stained tissues were blindly scored for histopathology based on the degree of epithelial damage pertaining to crypt architecture (normal, 0 - severe crypt distortion with loss of entire crypts, 3), degree of inflammatory cell infiltration (normal, 0 - dense inflammatory infiltrate, 3), muscle thickening (base of crypt sits on the muscularis mucosae, 0 - marked muscle thickening present, 3), goblet cell depletion (absent, 0- present, 1) and crypt abscess (absent, 0- present, 1) (Cooper et al., 1993). DAB staining was performed using HRP/DAB (ABC) Detection IHC Kit as per manufacturers protocol (ab64261, Abcam). Antibodies and staining protocol used are listed in **Supplementary Table 1**.

### Microscopy

Samples were imaged using the Zeiss Axio Scan.Z1 slide scanner or Zeiss LSM900 Confocal (Zeiss, Toronto, Canada). Crypt length and area measurements were collected using the Zen Blue Software (Zeiss). Images were processed using Adobe Photoshop (San Jose, CA). Antibody-positive cells were quantified as the total number of labeled cells or nuclei/crypt, area (for measurements combining crypts and lesions), Villin positive epithelial cells, or CD45 positive blood cells. Ten crypts were sampled per animal for each colon region (proximal and distal) for quantification of ChgA, Nr1d1, CD45, pHH3, cleaved caspase-3 and Cldn1. Regions were averaged together for results shown if no significant differences were observed. The Villin/Nr1d1 and Cldn1 stained tissue were scanned using the Zeiss LSM900 Confocal while all remaining samples were imaged using the Axio Scan.Z1.

### Per2::Luciferase Luminescence

*Per2::Luc* mice 14-15 weeks of age were euthanized and sections of 0.5cm of the proximal colon were collected in Hanks’ balanced salt solution supplemented with HEPES, NaHCO3, and penicillin-streptomycin. Proximal colon from untreated animals (n=5) or animals treated for 6 days with 4% DSS (n=6) were cleaned of adipose tissue and cultured on 0.4 mm membrane inserts (Millipore) in sealed 35 mm Petri dishes with 1.5 mL of DMEM supplemented with HEPES, NaHCO3, penicillin-streptomycin, B27 and beetle luciferin (Gold Biotechnologies). Bioluminescence rhythms were measured for at least 4 days with a luminometer (Actimetrics Inc) in a light-tight incubator set to 37°C, period and amplitude were calculated with LumiCycle (Actimetrics Inc) (Kloehn et al., 2016; Carmona-Alcocer et al., 2018).

### RNA Purification

A bullet blender was used to homogenize tissue samples (Next Advance, Troy, NY). RNA was isolated using the RNEasy mini Purification Kit (Qiagen). Prior to production of complimentary DNA, RNA was purified from the DSS *via* LiCl precipitation as described in (Viennois et al., 2018). LiCl purification was performed on all samples regardless of treatment (DSS and untreated). Complementary DNA was produced using the iScript RT Supermix (Bio-Rad) according to the manufacturer’s protocol.

### Quantitative Polymerase Chain Reaction

Transcriptional gene expression was analyzed using iTaq Universal SYBR Green Supermix (Bio-Rad) on a Viia7 real-time polymerase chain reaction plate reader (Thermo Fisher Scientific). Primer sequences were validated with an efficiency of approximately 80–110. The primers used are listed in **Supplementary Table 2**. Primer sequences were obtained from PrimerBank, Brooks et al. (2021), Kwon et al. (2019) and Yamazaki et al. (2011). All gene expression was normalized to *Gapdh*.

### Cytokine Protein Analysis

Using a whole tissue lysate of DSS treated (5 day, 5% w/v) colon tissue, levels of the pro-inflammatory cytokine TNF was determined by ELISA.

### RNAScope

RNA *in situ* probe hybridization and detection was performed using RNAScope protocol according to manufacturer’s instructions (Advanced Cell Diagnostics/Bio-Techne). The probes used were RNAscope Probe- Mm-Lgr5 ACDBio Cat# 312171 and RNAscope Probe- Mm-Hopx-C2 ACDBio Cat# 405161-C2. RNAScope slides were imaged and quantified by counting visible RNA positive puncta and averaging puncta per crypt or puncta per 5000μm^2^ in lesions.

### Statistical Analysis

Statistical analysis was performed using Prism 9 (Graphpad). Unpaired *t*-test, One-way ANOVA or Two-way ANOVA were used with Tukey’s multiple comparisons post-test; see **Supplementary Table 3** for all details. Rhythmic profile was evaluated with Chronos-Fit program (P. Zuther, S. Gorbey and B. Lemmer, Chronos-Fit 1.06, 2009).

## Results

### Characterization of the *Bmal1^-/-^* Mutant Colon Prior to Colitis

The *Bmal1* ^-/-^ mutant has been reported to have metabolic and age-related phenotypes (Kondratov and Antoch, 2007), but we have previously shown that the small intestinal epithelium is morphologically and cellularly normal in young adult mice (Stokes et al., 2017). In order to investigate pre-existing differences in the colon epithelium, *Bmal1^+/+^* control and *Bmal1^-/-^* mutant mice were collected at 13-15 weeks of age, before age-related phenotypes are apparent, and their colon epithelium was compared. The loss of *Bmal1* produced no obvious differences in tissue morphology (**Figure 1A**). The number of crypts in both proximal and distal regions of the colon, and average length of the crypts in these were indistinguishable between *Bmal1^+/+^* control and *Bmal1^-/-^* mutant genotypes, indicating no overt differences in cell proliferation/production (**Figures 1B, C**). We tested for changes in muscle thickness in the *Bmal1^+/+^* control and *Bmal1^-/-^* mutant colon, to determine whether inflammation is *Bmal1*-dependent under normal conditions, but this was equivalent in both genotypes (**Figure 1D**). To directly investigate cell proliferation, we stained tissue using antibody to Ki67, a proliferation marker that labels cells in S/G2/M phases; cell proliferation is equivalent between these genotypes (**Figures 1E, F**). Goblet cells produce a protective mucus in the intestine, and previous studies have shown that there are fewer goblet cells present in *Per1/2* mutant mice which predisposes these to colitis (Pagel et al., 2017). However, the *Bmal1^-/-^* mutant contains equal goblet cell numbers and the staining of mucin is similar to controls, suggesting no obvious mucus deficiency (**Figures 1G, H**). Enteroendocrine cells are another type of secretory cell which act as key sensors of metabolites (Karaki et al., 2006; Nøhr et al., 2013). We discovered a significant decrease in enteroendocrine cell presence, marked by Chromogranin A (O’Connor, 1983; Cetin et al., 1989) in *Bmal1^-/-^* mutant colon tissue (**Figures 1I, J**). Overall, our data shows that the colon epithelium does not appear to require *Bmal1* for epithelial cell production or tissue morphology in undamaged conditions but has a ~50% decrease in enteroendocrine cells.

### Colitis Dampens Circadian Rhythms in the Colon

Several reports have indicated that inflammation can lower circadian rhythms (Liu et al., 2017; Weintraub et al., 2020). We therefore tested to see whether circadian clock-driven rhythmic gene expression in the colon is present during colitis. *Per2::Luciferase* (*Per2::Luc*) mice, a protein reporter of the circadian clock that contains *Luciferase* fused to the *Per2* gene (Yoo et al., 2004), were treated with DSS to induce colitis. Colon tissue was then dissected from mice and colon tissue explants were tested for luminescence over a period of 4 days. This revealed that *Per2::Luc* DSS-treated colons exhibit dampened rhythmic luminescence readings compared to untreated controls (**Figure 2A**). DSS-treated *Per2::Luc* mice that are rhythmic show the same circadian period (**Figure 2B**), however the amplitude of these rhythms is much lower (**Figure 2C**) and half of DSS-treated mice do not show any rhythmicity (**Figures 2D, E**). When present, clock gene rhythms persist for several days in both controls and DSS-treated *Per2::Luc* mice, indicating that a free-running clock is present in the colon. These data suggest that clock transcriptional output in mice with colitis is weaker than normal, consistent with previous findings that show RT-qPCR rhythms in the DSS-treated colon are weaker (Wang et al., 2018).

The colon contains epithelial cells, but also many non-epithelial cells in the supporting lamina propria, surrounding muscle, blood vessels, and immune system. We further tested for circadian rhythms in epithelial and immune system cells at the protein level using histology sections. *Bmal1^+/+^* control and *Bmal1^-/-^* mutant mice were treated with DSS, and colon tissue was collected at three time points. We probed for Nr1d1 protein, the transcriptional target of *Bmal1*, to serve as a reporter of circadian clock output, and co-stained for Villin and/or CD45^+^ to distinguish the epithelial colon cells from blood-derived cells that are interspersed in this tissue (Pilling et al., 2009). The expression of Nr1d1 was evident in both Villin^+^ epithelial cells and CD45^+^ blood cells (**Figures 2F–H**). In control animals, the number of Nr1d1^+^ cells are increased in both cell populations at ZT20 with decreased expression earlier at ZT4 and ZT12 in both Villin^+^ epithelial cells (**Figure 2G**) and CD45^+^ blood cells (**Figure 2I**). However, this increase in Nr1d1 in controls is subtle, consistent with previous reports that have shown Nr1d1 expression is suppressed during colitis (Wang et al., 2018). Nr1d1 was noticeably decreased in *Bmal1^-/-^* mutant Villin^+^ epithelial cells (**Figure 2G**) and CD45^+^ blood cells (**Figure 2I**), with no significant daily rhythms in expression present (**Figures 2F–I**). These data suggest that *Bmal1*-dependent circadian rhythms are present in both the epithelial and blood cells in the colon during colitis. Together our data confirm that clock rhythms occur during colitis, and suggest that their weaker amplitude is not a result of desynchrony between colon epithelial and immune system cells.

### *Bmal1^-/-^* Mutant Mice Have More Severe Colitis and Increased Loss of Epithelial Structure

To investigate the effects which a non-functional circadian clock has on IBD, *Bmal1^+/+^* controls and *Bmal1^-/-^* mutants were treated with DSS, and their recovery tracked for an additional 10 days. We observed a drastic decrease in the recovery of *Bmal1^-/-^* mutant mice, indeed nearly all *Bmal1^-/-^* mutants are not able to tolerate colitis and had to be euthanized much earlier than controls (**Figure 3A**). This supports a critical role for *Bmal1* in IBD severity. We next tested if the increased disease severity of *Bmal1* mutants indicates that the colon epithelium of mice lacking a functional circadian clock is compromised. Colon tissue from *Bmal1^+/+^* controls and *Bmal1^-/-^* mutants were analyzed after DSS treatment. *Bmal1^-/-^* mutant mice exhibited a decrease in overall colon length (**Figures 3B, C**). This supports recent studies which have shown decreased colon length in Rorα intestinal epithelial cell conditionally knocked out mice following DSS treatment (Oh et al., 2019), and in a different *Bmal1^-/-^* mutant mouse strain than used in our current study (Wang et al., 2018). Pronounced morphological differences in *Bmal1^-/-^* mutant colon epithelia were readily apparent (**Figure 3D**). An increase in the size of lesions, regions of epithelium which are morphologically abnormal and contain no crypts or structure was observed in *Bmal1^-/-^* mutants (**Figure 3E**). Muscle tissue thickness, indicative of inflammation, was also increased in *Bmal1^-/-^* mutants (**Figure 3F**). Histopathological scores assessed in a double-blind analysis, confirmed that *Bmal1^-/-^* mutant colon tissue exhibits increased muscle thickening (**Figure 3G**). Loss of architecture in *Bmal1^-/-^* mutant colon was also noted (**Figure 3H**), consistent with the previously mentioned increase in lesion size present in *Bmal1^-/-^* mutants. Additionally, immune cell infiltration was increased in *Bmal1^-/-^* mutant colons (**Figure 3I**). Although no differences were seen in goblet cell depletion and crypt abscess scores (**Supplemental Figure 1A, B**), together these features lead to increased overall histology score in *Bmal1^-/-^* mice with colitis (**Figure 3J**). These results demonstrate that loss of colon epithelial structure underlie the increased colitis observed in Bmal1 mutant mice.

The circadian clock has recently been shown to play a role in the rhythms of the microbial response, and the Antimicrobial Peptide (AMP) gene expression of both *Reg3g* and *Lcn2* exhibits diurnal rhythms (Bellet et al., 2013; Brooks et al., 2021). We therefore tested the expression of AMPs in untreated mice and mice treated with DSS to induce colitis (all samples collected at ZT16, when *Reg3g* and *Lcn2* have been shown to be higher). Both *Lcn2* and *Reg3g* exhibited significantly increased expression in *Bmal1^+/+^* control colon tissue following induction of DSS-colitis (**Figures 4A, B**). However, *Lcn2* and *Reg3g* expression in DSS-treated *Bmal1^-/-^* mutant tissue remains low. No significant differences were detected between *Bmal1^+/+^* controls and *Bmal1^-/-^* mutants in the expression of the *S100a8* AMP, or the anti-microbial enzyme *Lyz1* (**Figures 4C, D**), although, the expression of *S100a8* increased during colitis in both *Bmal1^+/+^* controls and *Bmal1^-/-^* mutants. These data suggest that certain mechanisms of anti-microbial activity are compromised during colitis in *Bmal1^-/-^* mutants.

To further investigate the colon epithelial integrity in the absence of clock function, genes involved in barrier function were measured. Previous studies have indeed shown that genes encoding the tight junction proteins Claudin1 (Cldn1) and Occludin (Zo1) are expressed in a daily rhythm in wildtype mice (Oh-oka et al., 2014). Since increased barrier permeability plays an important role in IBD, we stained for Cldn1 in *Bmal1^+/+^* controls and *Bmal1^-/-^* mutant tissue with colitis (**Figure 4E**). No differences in Cldn1 were apparent. We also tested the transcriptional expression of *Cldn1*, *Zo1*, *Tight junction protein 1 (Tjp1)* and *Mucin 2 (Muc2)*, however, no differences were detected (**Figures 4F–I**). These data suggest that the *Bmal1^-/-^* mutant does not have an obvious barrier phenotype.

### Diurnal Rhythms in Inflammatory and Regenerative Processes Are Lost in Circadian-Deficient *Bmal1^-/-^* Mice

If circadian rhythms have a regulatory role in colitis, disease severity should vary depending on time of day. In order to monitor the progression of inflammatory bowel disease, a disease activity score was assessed based on the severity of individual weight loss, blood presence in stool, softening of stool or diarrhea, and rectal bleeding (Kim et al., 2012). Disease activity was tested daily at ZT0 and ZT8 to assess differences in colitis severity at different times over a period of five days. ZT0 is the early morning, the end of nocturnal mouse activity, and ZT8 is just past midday, the middle of the rest phase where regenerative processes are thought to occur. When scored at ZT0 *Bmal1^+/+^* controls show significantly lower colitis than *Bmal1^-/-^* mutants particularly in stool consistency (diarrhea) (**Figure 5A**) and fecal blood (**Figure 5B**), leading to increased total disease severity (**Figure 5C**) despite having no differences in weight loss (**Supplementary Figure 2A**). These differences are not present later in the day at ZT8. Instead, disease scores for *Bmal1^+/+^* were increased to levels that were not significantly different from *Bmal1^-/-^* mutants (**Figures 5D–F** and **Supplementary Figure 2B**). We then re-analyzed *Bmal1^+/+^* disease scores and noted that stool consistency (diarrhea) is more severe at ZT8 driving an overall higher total disease score if mice were examined later in the day (**Figures 5G–I** and **Supplementary Figure 2A, B**). Notably, *Bmal1^-/-^* mutants remained at the same high levels at both times examined (no significant differences in **Figures 5A–F**). These results suggest that colitis exhibits time-dependent disease activity in *Bmal1^+/+^* circadian-competent animals, with increased severity during rest (ZT8). Rhythms in disease severity in *Bmal1^-/-^* clock-deficient animals are not present and instead disease activity is consistently high at different times of day.

Daily changes in disease activity suggest underlying changes of cellular activity in the colon leading to increased inflammation. Thus, we investigated the pro-inflammatory cytokine expression in *Bmal1* ^+/+^ control and *Bmal1^-/-^* mutant colon tissue following DSS-colitis. While no differences are present in *IL-1β*, and *IL-6*, an increase in *TNF* expression was detected in *Bmal1^-/-^* mutant colon tissue at ZT4 (**Figures 6A–**C). Two additional cytokines, *IL-10* and *IL-22*, did not show a difference between genotypes (data not shown). Given that the change in *TNF* expression occurs at the onset of rest (for mice), we collected *Bmal1* ^+/+^ control and *Bmal1^-/-^* mutant colon tissue at ZT0 and measured TNF colonic protein concentrations. TNF protein is higher in the *Bmal1^-/-^* mutant colon suggesting an increase in inflammation (**Figure 6D**).

Next, we examined histological samples and determined cellular infiltration histopathological scores over 24 hours (ZT0, 4, 8, 12, 16, and 20). Cellular infiltration in the colon, by score, does not vary over time in either *Bmal1^+/+^* controls or *Bmal1^-/-^* mutants (**Figure 6E**), however *Bmal1^-/-^* mutants have significantly higher cellular infiltration scores overall (**Figure 3I**). To further test this, we probed the same cohort of *Bmal1^+/+^* control and *Bmal1^-/-^* mutant mice for immune system blood cells (marked by anti-CD45) (Pilling et al., 2009) which infiltrate tissue lesions during DSS-induced colitis (**Figure 6F**). We examined both lesioned regions and morphologically normal regions to determine if a difference was present. When the colonic lamina propria (LP) was examined, no differences in the daily timing of CD45^+^ cellular presence was evident in either *Bmal1^+/+^* and *Bmal1^-/-^* mice (**Figure 6G**). Reanalyzing these data by averaging CD45^+^ number irrespective of time shows that an increase in CD45^+^ cellular infiltration is present in the *Bmal1^-/-^* mutants (**Figure 6H**). This is consistent with the increased cellular infiltration histology score (compare **Figure 6E** and **Figure 3G**). We then looked specifically at lesioned tissue in the DSS-treated colons to test the immune cell response in areas of greater injury. A significant increase in CD45^+^ cells at ZT12, the time when mice normally become active, was observed in control *Bmal1^+/+^* animals relative to times of rest (**Figure 6I**). This daily change in blood cell numbers is not present in *Bmal1^-/-^* mutant tissue who have low levels of CD45^+^ cellular infiltration at these same times. We conclude that controls show an increase of immune cell presence in DSS-damaged tissue that peaks at the same time as activity usually begins, but these changes are absent in circadian-deficient *Bmal1^-/-^* mice that show elevated inflammation throughout the colon.

The epithelial barrier is damaged during IBD and is required to ultimately resolve inflammation. We tested the epithelial response during DSS-treatment, to determine if *Bmal1^-/-^* mutants have defects in regeneration. We focused on the non-ulcerated regions of the colon where crypt structures containing stem and precursor cells persist during colitis. Substantial proliferation differences (marked by Ki67) are evident in *Bmal1^+/+^* control mice based on time of day, while proliferation is consistently low in *Bmal1^-/-^* mutants (**Figures 7A, B**). This contrasts with crypts prior to the induction of colitis, where there is no difference in proliferation (**Figures 1E, F**). We further tested proliferation using a cohort of *Bmal1^+/+^* control and *Bmal1^-/-^* mutant mice treated with DSS, collected at 6 time points during the entire 24-hours. Diurnal rhythms in colon epithelial mitosis (marked by phosphorylated-Histone-H3; pHH3) are present in *Bmal1^+/+^* controls throughout the day, with a peak in mitosis taking place around ZT0 at the onset of rest (**Figures 7C, D**). These rhythms are completely disrupted and decreased in *Bmal1^-/-^* mutants. Of note, we also tested for epithelial cell apoptosis in the same samples (marked by activated Caspase-3; Cas3) but no differences are apparent in a time- or genotype-dependent manner (**Figures 7E, F**). The Ki67 and pHH3 data suggest that daily proliferation rhythms during colitis are present in the epithelium of *Bmal1^+/+^* controls and are suppressed in circadian-deficient *Bmal1^-/-^* mutants. These data indicate that the regenerative ability of the colon follows daily rhythms that are opposite to those observed in blood cell activity (**Figure 6**), peaking at the end of the active period rather than the onset of activity. Like colitis symptoms (**Figure 5**), these rhythms are not present in *Bmal1^-/-^* mutant mice.

### Regeneration Requires *Bmal1*

The colon epithelium is regenerated by stem cells and precursors that replenish cells lost during infection or injury (Yui et al., 2018). We probed for markers of these cells in the crypts of the colon epithelium prior to DSS-treatment, during DSS-induced colitis, and following a 10-day recovery period (**Figure 8A**). RNA probes were used to identify intestinal stem cells, marked by *Lgr5*, that renew the colon daily (Barker et al., 2007), as well as fetal-like precursors marked by *Hopx*, involved in the colitis response (Takeda et al., 2011; Wang et al., 2019). In the untreated colon, the expression of *Hopx* and *Lgr5* is significantly weaker in the *Bmal1^-/-^* mutant epithelium (**Figures 8B–D**). This is also apparent upon DSS-induced colitis where the expression of *Hopx* and *Lgr5* remains higher in the morphologically intact proliferating crypts of *Bmal1^+/+^* controls at 6 days but is decreased in *Bmal1^-/-^* mutant animals (**Figures 8E–G**). Of note, in the lesions present in the same DSS-injured colon tissues *Hopx* is decreased in *Bmal1^-/-^* mutants (**Figures 8H, I**), but *Lgr5* expression is reduced in both *Bmal1^+/+^* control and *Bmal1^-/-^* mutant lesions (**Figure 8J**). These data are consistent with a recent study (Girish et al., 2021) which has shown *Lgr5* is sensitive to DSS treatment. Finally, following the 10-day recovery period, *Hopx* expression is similar in both genotypes but *Lgr5* expression remains significant reduced in *Bmal1^-/-^* mutants (**Figures 8K–M**). At this stage of recovery, *Bmal1^+/+^* controls no longer have any DSS-induced lesions present (colitis is resolved), but *Hopx* and *Lgr5* expression persists in the *Bmal1^-/-^* mutant lesions (**Figures 8N–P**). Overall, these data suggest the expression of stem cell and precursor cell markers is reduced in *Bmal1^-/-^* mutants at all stages pre- and post-colitis. We note that these timelines are generally consistent with previous findings (Harnack et al., 2019; Wang et al., 2019), but vary slightly by time perhaps due to the mouse genetic background and/or different levels of DSS used in our study.

To further assess the regenerative ability of mice without a functional circadian clock, we examined morphological differences between *Bmal1^+/+^* controls and *Bmal1^-/-^* mutants following 10 days of recovery (**Figure 9A**). An increase in lesions is apparent in *Bmal1^-/-^* colon tissue, who cannot resolve colitis. In contrast, nearly all lesions in control animals that were present (see **Figure 3E**) are now resolved and crypt architecture has returned to normal (**Figure 9B**). However, large lesions continue to be present in *Bmal1^-/-^* mutants well into recovery (**Figure 9B**), and a corresponding decrease in the crypt density of *Bmal1^-/-^* colons is evident (**Figure 9C**). In the non-lesioned parts of the colon, *Bmal1^-/-^* mutants also exhibit an increased average crypt length reminiscent of the hyperplasia associated with IBD (Erben et al., 2014) (**Figure 9D**). An increase in average muscle thickness is also present in *Bmal1^-/-^* colon tissue following 10 days of recovery (**Figure 9E**), similar to that noted during colitis (**Figure 3F**). These results indicate that the regenerative abilities of *Bmal1^-/-^* mutants are significantly impaired.

## Discussion

Using a mouse model of circadian disruption *via Bmal1*, we have shown that the absence of circadian rhythms leads to more severe colitis, a form of IBD. During colitis, circadian clock activity is present in the epithelial and blood cells of *Bmal1^+/+^* controls although the rhythms have lower amplitude (**Figure 2**), meaning that the rhythms are weaker in their transcriptional strength and thus functional output. Both epithelial and blood cells in the colon show daily patterns of cellular activity, that are completely absent in *Bmal1^-/-^* mutants (**Figures 6**, **7**). This suggests that rhythms in the cells of the colon play an important role in resolving colitis. Indeed, we discovered that symptoms of colitis change over the course of a day (**Figure 5**) and that the loss of these rhythms is detrimental (**Figure 3**). Loss of *Bmal1* also reduces the expression of AMPs (**Figure 4**), and increases inflammation (**Figure 6**), suggesting that certain aspects of the colitis response is absent. Our data demonstrate that circadian rhythms are a feature of colitis and that the core clock gene, *Bmal1*, is critical in resolving colitis. Our data using the *Bmal1^-/-^* mutant are consistent with a recent study that found DSS-induced colitis is increased in severity in a different *Bmal1* mutant mouse strain, with higher disease scores and poor recovery compared to controls (Wang et al., 2018).

We have built from these findings and determined that in addition to affecting colitis progression, *Bmal1* promotes the regeneration of the colonic epithelium. A significant decrease in cell proliferation occurs in *Bmal1^-/-^* mutants (**Figure 7**), consistent with proliferation rhythms that we have previously observed in the small intestine during the Gastrointestinal Syndrome (Stokes et al., 2017), and in the *Drosophila* intestine treated with DSS (Karpowicz et al., 2013). This suggests the clock is a fundamental regulator of regenerative timing throughout the digestive tract of animals (Parasram and Karpowicz, 2020). In *Bmal1^-/-^* mutants, a decrease in *Lgr5* and *Hopx* expression in the colon epithelium indicates that regenerative pathways are disrupted (**Figure 8**). Since both Wnt pathways (*via* intestinal stem cells) and Hippo pathways (*via* Hopx^+^ fetal-like precursors) contribute to post-colitis regeneration (Harnack et al., 2019; Wang et al., 2019), the clock may function to positively regulate healing through the activity of these stem and precursor cells. Indeed, *Bmal1* mutant mice carry a greater burden of ulcers/lesions throughout the colon (**Figure 3**), and these do not resolve after colitis whereas those of controls are completely healed (**Figure 9**). This is consistent with *Bmal1* acting as a positive regulator of tissue regeneration. Of note, prior to DSS-treatment, *Bmal1^+/+^* control *Bmal1^-/-^* mutant colon morphologies are relatively unchanged (**Figure 1**), suggesting that *Bmal1* functions during acute healing phases but the body can compensate in its absence during general cellular turnover. We do, however, see decreased populations of enteroendocrine cells present in *Bmal1^-/-^* mutant colon which may impair hormone secretion in the inflammatory response (Worthington, 2015). Future research will determine whether and why enteroendocrine cells are reduced when the circadian clock is disrupted.

It has been well-established that the immune system and the infection response have circadian rhythms (Curtis et al., 2014; Man et al., 2016; Scheiermann et al., 2018). Inflammatory responses from immune system cells are also known to regulate regenerative responses in the intestinal epithelium (Pickert et al., 2009; Lindemans et al., 2015; Taniguchi et al., 2015; Jeffery et al., 2017; Romera-Hernández et al., 2020). However, the effect of circadian rhythms on IBD is still an emerging field. The role of clock genes as immuno-regulators of colitis is supported by three recent studies (Pagel et al., 2017; Wang et al., 2018; Oh et al., 2019), as well as our present work where we demonstrate increased inflammation during resting periods in *Bmal1^-/-^* mutant mice. The clock component, *Nr1d1*, has been shown to repress the inflammasome *via* the NF-κB/Nlrp3 pathway (Wang et al., 2018). In *Nr1d1* mutants treated with DSS, inflammation is exacerbated leading to increased DSS-induced colitis. In the *Bmal1^-/-^* mutant, *Nr1d1* expression is lower so this pathway likely also contributes to the phenotypes we observe. *Rorα*, another circadian clock gene, has a role in repressing NF-κB pathway activation thereby suppressing the inflammatory response (Oh et al., 2019). In mice with an intestinal epithelial-specific knockout of *Rorα* DSS-induced colitis is also increased. These studies reveal that *Nr1d1* and *Rorα* genes, that are both part of the secondary arm of the circadian clock, are repressors of inflammatory colitis. However, these genes are redundant in terms of clock function, hence the colitis phenotypes of the mutants are most likely attributable to *Nr1d1* and *Rorα* function rather than a result of circadian rhythm loss which persists in the mouse strains used in these studies. Mice with a *Per1;Per2* double knockout exhibit reduced mucous barrier function and increased cellular death when treated with DSS (Pagel et al., 2017). This closely resembles our current work because both the *Per1;Per2* double mutant and the *Bmal1^-/-^* mutant have dysfunctional circadian clock activity and hence loss of physiological rhythms (Bunger et al., 2000; Zheng et al., 2001). In *Per1;Per2* mice given DSS, increased cytokine levels, worsened histopathology, and decreased overall recovery are strikingly similar to the results we report in *Bmal1^-/-^* mutant mice. Pagel et al. additionally demonstrated impaired epithelial cell proliferation like we observed in the *Bmal1^-/-^* mutants (**Figure 7**). Our study therefore adds to a growing body of literature that supports the role of the circadian clock in mediating IBD severity through inflammatory and regenerative processes.

Our primary objective in the present study was to determine whether colitis pathology, or its underlying cellular mechanisms, have daily rhythms. We find that the colon indeed exhibits daily rhythms in disease activity (**Figure 5**), immune cell presence (**Figure 6**) and epithelial regeneration (**Figure 7**) during colitis. These rhythms are disrupted in *Bmal1^-/-^* clock non-functional mice, demonstrating these cellular processes are regulated by the circadian clock. The timing of colitis and immune cell infiltration into lesions (**Figures 5**, **6)**, that is higher at midday (ZT8-12) before activity begins, is opposed to the timing of cell proliferation (**Figure 7**), that is higher in the morning (ZT0) when activity ends. Based on these findings, we propose that circadian clock in the colon regulates the timing of the gastrointestinal regenerative response to optimize it with respect to other aspects of gastrointestinal physiology. Studies have shown that disruptions to the photoperiod itself can augment DSS-induced colitis in circadian-competent mice (Preuss et al., 2008; Amara et al., 2019). It is thus possible that the loss of rhythmic physiological timing is sufficient to recapitulate the same phenotypes we observe in mutant mouse strains. Future work is needed to confirm this at the cellular level, and to precisely resolve the underlying cell biology and the molecular timing of the cross-talk between the epithelium and the immune system during colitis. Indeed, recent findings have shown that *Bmal1* functions in macrophages (Wang et al., 2018) and B-cells (Liu et al., 2021) during colitis to resolve inflammation. Because the inflammatory response is closely intertwined with the regenerative response (Karin and Clevers, 2016), these findings are consistent with our data that show regenerative processes are also disrupted in *Bmal1* mutants. The circadian clock has arisen as a temporal regulator of physiology, and therefore it is possible that it times the healing process in the immune-epithelial cell axis to separate regeneration from digestion, metabolism, and immune system activity. Future studies that test the effects of desynchronizing these physiological processes in respect to each other will determine whether and how the loss of rhythms in the colon are detrimental during colitis.

In addition to being subject to the activity of neighboring immune system cells, the colon epithelium also interacts with its resident microbiome (Tognini et al., 2017; Choi et al., 2021). Recent studies have shown that the intestinal microbiome elicits a transcriptional response (Thaiss et al., 2016), including profound epigenetic changes (Kuang et al., 2019), to promote colon health (Thaiss et al., 2014; Wang et al., 2017). It is thus likely that in *Bmal1^-/-^* mutant mice, where these processes are disrupted, the microbiome contributes to colitis severity. Indeed, gastrointestinal infection of *Salmonella* is time- and clock-dependent (Bellet et al., 2013), suggesting that microbial dysbiosis in the context of IBD is likely to play a role in the rhythmic inflammation and regeneration outcomes that we observe. It has been shown that diurnal rhythms in AMP production are a component of the gut’s microbial interactions (Brooks et al., 2021), and our data support anti-microbial mechanisms as an important circadian factor in IBD (**Figure 4**). An additional factor is sex, which has been shown to regulate male *versus* female circadian rhythms in microbial responses that contribute to gastrointestinal health (Liang et al., 2015; Weger et al., 2019). Future studies investigating these complex relationships between the circadian clock, sex, the microbiome, and colon tissue cells will address these precise connections. Our work shows that the colon epithelium is key in understanding the circadian biology of this dynamic tissue.

Our results are not only significant in developing animal models for IBD research but may be translatable in a clinical setting. Patients with IBD often complain about loss of sleep (Ranjbaran et al., 2007), and regular sleep disturbance can disrupt circadian rhythms. Indeed, sleep loss has been shown to increase the inflammatory response *via* over activation of NF-κB (Irwin et al., 2008). We provide additional evidence that circadian disruption leads to increased disease severity of colitis. As IBD symptoms worsen, patients experience increased sleep disturbance, this may result in a self-perpetuating feedback cycle which propels disease progression. Our findings are also significant in IBD diagnostics. Given that patient visits are likely to be scheduled during the day, IBD symptoms are typically assessed during our physiological active hours. However, our results using nocturnal mice show that disease severity exhibits daily rhythms which is increased during the end of physiological activity (*i.e.* late evening in humans). As a result, diagnostics which are carried out during the early daytime might miss the severe symptoms. Our work and that of others provide important preventative measures for patients with IBD to avoid shift work which would disrupt their natural 24-hour rhythms and worsen overall disease progression. This may aid in managing IBD in patients. Finally, appreciating that colitis is time-dependent could also enhance the effectiveness of pharmaceutical treatments based on the times of the day during which inflammation/regeneration vary.

## Data Availability Statement

The raw data supporting the conclusions of this article will be made available by the authors, without undue reservation.

## Ethics Statement

The animal study was reviewed and approved by University of Windsor, ACC.

## Author Contributions

ZT, VC-A, KS, MH, and HW contributed to experiments and experimental analysis. SC, WK, and PK contributed to oversight of project and research planning and interpretation. ZT, VC-A, and PK contributed to analysis and writing. All authors contributed to the article and approved the submitted version.

## Author Disclaimer

The contents of this article are solely the responsibility of the authors and may not represent the official views of the sponsoring agencies.

## Conflict of Interest

The authors declare that the research was conducted in the absence of any commercial or financial relationships that could be construed as a potential conflict of interest.

## Publisher’s Note

All claims expressed in this article are solely those of the authors and do not necessarily represent those of their affiliated organizations, or those of the publisher, the editors and the reviewers. Any product that may be evaluated in this article, or claim that may be made by its manufacturer, is not guaranteed or endorsed by the publisher.

## Supplementary Material

Supplementary Figure 1 Additional tests of histopathological severity. **(A)** No difference in goblet cell depletion scores between *Bmal1* ^+/+^ control and *Bmal1^-/-^* mutant mice with colitis were noted (Unpaired *t*-test, p=0.3429). **(B)** No difference in crypt abscess between genotypes were observed (Unpaired *t*-test, p=0.9734). (All error bars represent SEM).

Supplementary Figure 2 Additional tests of disease severity. No differences in weight loss were found between *Bmal1* ^+/+^ control and *Bmal1^-/-^* mutant mice when examined at **(A)** ZT0 (Two-way ANOVA, p=0.2789, F_(2,25)_=1.351; Tukey’s test, ns), or at **(B)** ZT8 (Two-way ANOVA, p=0.0577, F_(4,30)_=2.575; Tukey’s test, ns). (All error bars represent SEM).

Supplementary Table 1 Summary of Antibodies and Staining Protocols.

Supplementary Table 2 List of Primers Sequences.

Supplementary Table 3 Summary of Statistical Analyses.
