# Supplementary figures and images for: BMAL1 Regulates the Daily Timing of Colitis
Source: Front Cell Infect Microbiol. 2022 Feb 9;12:773413. doi: 10.3389/fcimb.2022.773413 (PMC8863668; doi:10.3389/fcimb.2022.773413)

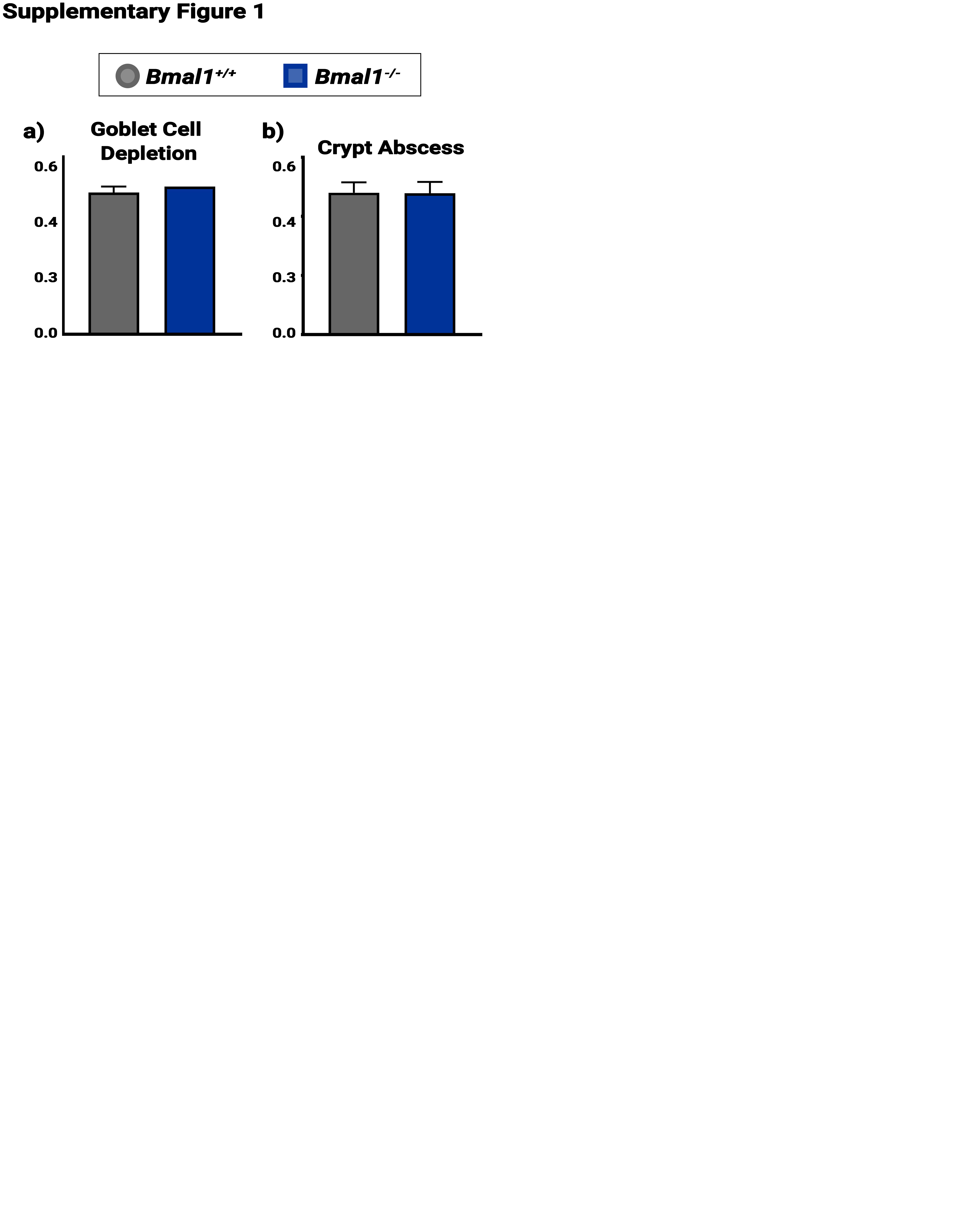

Supplement: Supplementary Figure 1 — Additional tests of histopathological severity. (A) No difference in goblet cell depletion scores between Bmal1 +/+ control and Bmal1-/- mutant mice with colitis were noted (Unpaired t-test, p=0.3429). (B) No difference in crypt abscess between genotypes were observed (Unpaired t-test, p=0.9734). (All error bars represent SEM). [file Image_1.jpeg]

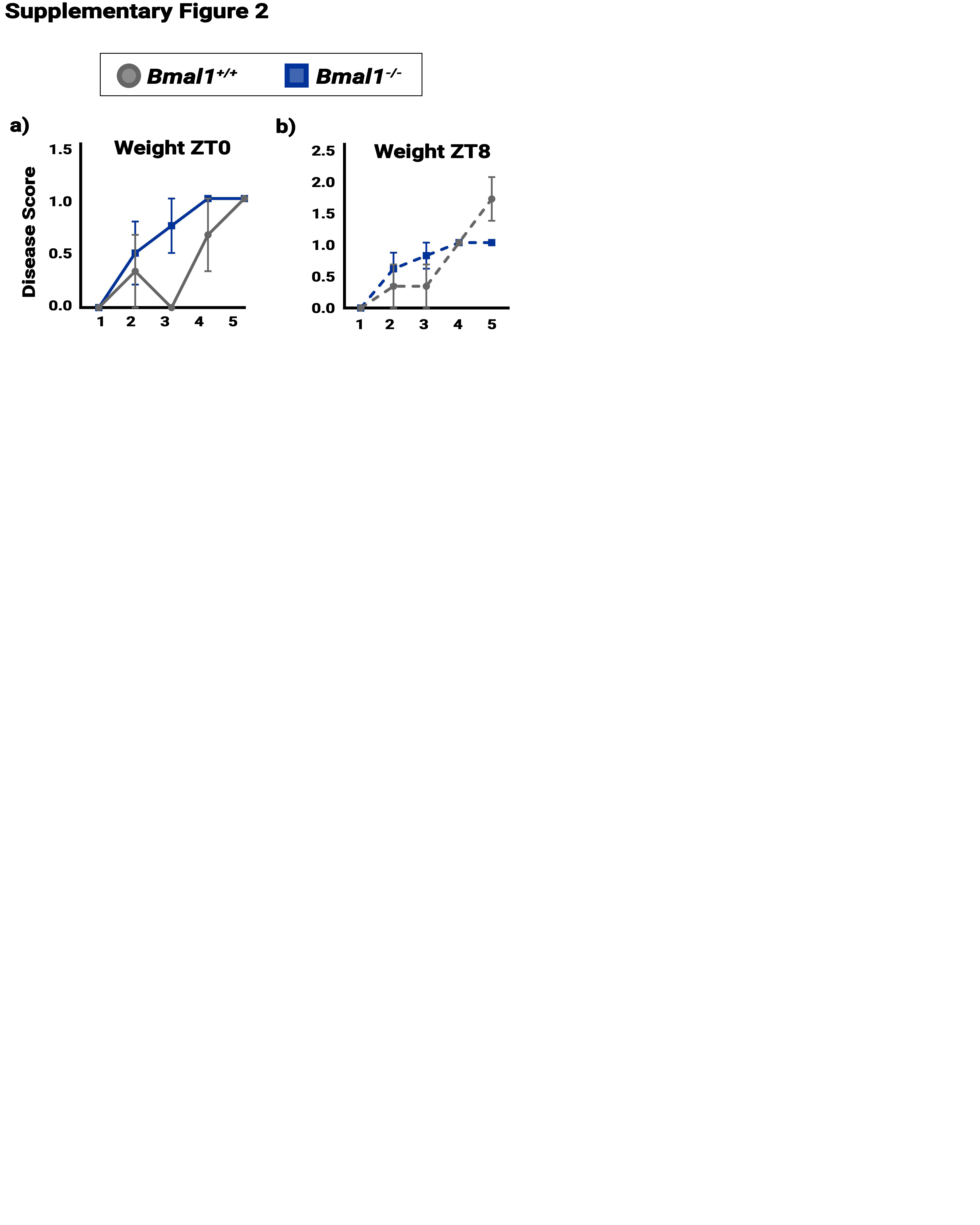

Supplement: Supplementary Figure 2 — Additional tests of disease severity. No differences in weight loss were found between Bmal1 +/+ control and Bmal1-/- mutant mice when examined at (A) ZT0 (Two-way ANOVA, p=0.2789, F(2,25)=1.351; Tukey’s test, ns), or at (B) ZT8 (Two-way ANOVA, p=0.0577, F(4,30)=2.575; Tukey’s test, ns). (All error bars represent SEM). [file Image_2.jpeg]
