# Supplementary material for: BMAL1 Regulates the Daily Timing of Colitis
Source: Front Cell Infect Microbiol. 2022 Feb 9;12:773413. doi: 10.3389/fcimb.2022.773413 (PMC8863668; doi:10.3389/fcimb.2022.773413)
Supplement: Supplementary Table 1 — Summary of Antibodies and Staining Protocols. [file Table_1.docx]

**Supplementary Table 1. Summary of Antibodies and Staining Protocols.**

| **Antibody** | **Product Info** | **Dilution** | **Protocol** | **Blocking Agent** | **Antigen Retrieval** |
| --- | --- | --- | --- | --- | --- |
| CD45 | Abcam  Ab10558 | 1:200 | IF | 1% Milk in PBS | Sodium Citrate 95˚C water bath,  30 min |
| Chromogranin A  (ChgA) | Abcam Ab45179 | 1:100 | IF | 5% NGS in PBST | 100% Methanol,  5 min |
| Claudin1 | Abcam Ab211737 | 1:250 | IF | 5% NGS in PBS | Sodium Citrate 90˚C water bath,  20 min |
| Cleaved Caspase 3  (Cas3) | Cell Signaling Tech 9661S | 1:200 | IF | 5% NGS in PBST | - |
| Ki67 (DAB) | Abcam Ab16667 | 1:100 | DAB | 5% NGS in PBST | Sodium Citrate 90˚C water bath,  10 min |
| Nr1d1 | Abnova H00009572M02 | 1:250 | IF | 1% Milk in PBS | Sodium Citrate 95˚C water bath,  30 min |
| phospho Histone H-3 (Ser10) (pHH3) | Upstate/ Millipore  06-570 | 1:1000 | IF | 5% NGS in PBST | - |
| Villin | Abcam Ab130751 | 1:250 | IF | 1% Milk in PBS | Sodium Citrate 95˚C water bath,  30 min |
