# Supplementary material for: BMAL1 Regulates the Daily Timing of Colitis
Source: Front Cell Infect Microbiol. 2022 Feb 9;12:773413. doi: 10.3389/fcimb.2022.773413 (PMC8863668; doi:10.3389/fcimb.2022.773413)
Supplement: Supplementary Table 2 — List of Primers Sequences. [file Table_2.docx]

**Supplementary Table 2. List of Primers Sequences.**

| **Primer Name** | **Forward** | **Reverse** | **Source** |
| --- | --- | --- | --- |
| *Lcn2* | TGGCCCTGAGTGTCATGTG | CTCTTGTAGCTCATAGATGGTGC | PrimerBank |
| *Reg3g* | ATGCTTCCCCGTATAACCATCA | ATGCTTCCCCGTATAACCATCA | PrimerBank |
| *S100a8* | TGTCCTCAGTTTGTGCAGAATATAAA | TCACCATCGCAAGGAACTCC | (Brooks et al., 2021) |
| *Lyz1* | GTCACACTTCCTCGCTTTCC | TGACTGTCACCAGCATCCAT | (Brooks et al., 2021) |
| *Cldn1* | CTGGAAGATGATGAGGTGCAGAAGA | CCACTAATGTCGCCAGACCTGAA | (Yamazaki et al., 2011) |
| *Zo1* | ATGTCCGGCCGATGCTCTCTC | CTTTGGCTGCTCTTGGGTCTGTAT | (Kwon et al., 2019) |
| *Tjp1* | GCCGCTAAGAGCACAGCAA | TCCCCACTCTGAAAATGAGGA | PrimerBank |
| *Muc2* | GCTGACGAGTGGTTGGTGAATG | GATGAGGTGGCAGACAGGAGA | (Brooks et al., 2021) |
| *IL-1β* | TGGAGAGTGCTGTGGAAGAA | TGTGATGTACTGCTGAACCCT | PrimerBank |
| *IL-6* | TAGTCCTTCCTACCCCAATTTCC | TTGGTCCTTAGCCACTCCTTC | PrimerBank |
| *IL-22* | ATGAGTTTTTCCCTTATGGGGAC | GCTGGAAGTTGGACACCTCAA | PrimerBank |
| *TNF* | CCCTCACACTCAGATCATCTTCT | GCTACGACGTGGGCTACAG | PrimerBank |
| *IL-10* | GCTCTTACTGACTGGCATGAG | CGCAGCTCTAGGAGCATGTG | PrimerBank |
| *Gapdh* | AGGTCGGTGTGAACGGATTTG | TGTAGACCATGTAGTTGAGGTCA | PrimerBank |
