# Supplementary material for: BMAL1 Regulates the Daily Timing of Colitis
Source: Front Cell Infect Microbiol. 2022 Feb 9;12:773413. doi: 10.3389/fcimb.2022.773413 (PMC8863668; doi:10.3389/fcimb.2022.773413)
Supplement: Supplementary Table 3 — Summary of Statistical Analyses. [file Table_3.docx]

**Supplementary Table 3. Summary of Statistical Analyses.**

| **Fig.** | **Statistical Test** | **Post Hoc Test** | **Variable** | ***n*** | **Mean** | **± SEM** | ***p*** | ***F*** |
| --- | --- | --- | --- | --- | --- | --- | --- | --- |
| 1B | Unpaired *t*-test |  | *Bmal1^+/+^* |  | 30.01 |  |  |  |
|  |  |  | *Bmal1^-/-^* | 3 (each) | 29.16 | 1.805 | 0.6627 |  |
| 1C | Unpaired *t*-test |  | *Bmal1^+/+^* |  | 133.4 |  |  |  |
|  |  |  | *Bmal1^-/-^* | 3 (each) | 149.5 | 20.69 | 0.4786 |  |
| 1D | Unpaired *t*-test |  | *Bmal1^+/+^* |  | 125 |  |  |  |
|  |  |  | *Bmal1^-/-^* | 3 (each) | 132.6 | 28.47 | 0.8027 |  |
| 1F | Unpaired *t*-test |  | *Bmal1^+/+^* | 4 | 14.94 |  |  |  |
|  |  |  | *Bmal1^-/-^* | 5 | 11.99 | 2.263 | 0.2334 |  |
| 1H | Unpaired *t*-test |  | *Bmal1^+/+^* | 3 | 17.1 |  |  |  |
|  |  |  | *Bmal1^-/-^* | 3 | 20.6 | 1.922 | 0.1427 |  |
| 1J | Unpaired *t*-test |  | *Bmal1^+/+^* |  | 0.9192 |  |  |  |
|  |  |  | *Bmal1^-/-^* | 5 (each) | 0.4114 | 0.1033 | *0.0012 |  |
| 2B | Unpaired *t*-test |  | Untreated | 5 | 25.96 |  |  |  |
|  |  |  | DSS | 3 | 24.04 | 1.713 | 0.3131 |  |
| 2C | Unpaired *t*-test |  | Untreated | 5 | 584.1 |  |  |  |
|  |  |  | DSS | 3 | 67.84 | 188.7 | *0.0410 |  |
| 2G | One-way ANOVA |  | ZT4, 12, 20 *Bmal1^+/+^* | 3 (each) |  |  | *0.0383 | 5.828 |
|  | Two-way ANOVA |  | ZT4, 12, 20 *Bmal1^+/+^* |  |  |  |  |  |
|  |  |  | ZT4, 12, 20 *Bmal1^-/-^* | 3 (each) |  |  | *0.0083 | 7.333  (2, 12) |
|  |  | Tukey's test (*Bmal1^+/+^- Bmal1^-/-^*) | ZT4 |  |  |  | 0.8299 |  |
|  |  |  | ZT12 |  |  |  | 0.2814 |  |
|  |  |  | ZT20 |  |  |  | *0.0134 |  |
| 2I | One-way ANOVA |  | ZT4, 12, 20 *Bmal1^+/+^* | 3 (each) |  |  | *0.0483 | 5.238 |
|  | Two-way ANOVA |  | ZT4, 12, 20 *Bmal1^+/+^* |  |  |  |  |  |
|  |  |  | ZT4, 12, 20 *Bmal1^-/-^* | 3 (each) |  |  | *0.0437 | 4.111  (2, 12) |
|  |  | Tukey's test (*Bmal1^+/+^- Bmal1^-/-^*) | ZT4 |  |  |  | 0.6107 |  |
|  |  |  | ZT12 |  |  |  | *0.0391 |  |
|  |  |  | ZT20 |  |  |  | *0.0011 |  |
| 3A | Log-rank (Mantel-Cox) test |  | *Bmal1^+/+^* | 29 |  |  |  |  |
|  |  |  | *Bmal1^-/-^* | 21 |  |  | *<0.0001 |  |
| 3C | Unpaired *t*-test |  | *Bmal1^+/+^* | 5 | 6.92 |  |  |  |
|  |  |  | *Bmal1^-/-^* | 4 | 5.825 | 0.3841 | *0.0247 |  |
| 3E | Unpaired *t*-test |  | *Bmal1^+/+^* | 18 | 2.429 |  |  |  |
|  |  |  | *Bmal1^-/-^* | 20 | 3.984 | 0.6872 | *0.0298 |  |
| 3F | Unpaired *t*-test |  | *Bmal1^+/+^* | 16 | 139.9 |  |  |  |
|  |  |  | *Bmal1^-/-^* | 16 | 174.6 | 14.13 | *0.0201 |  |
| 3G | Unpaired *t*-test |  | *Bmal1^+/+^* | 25 | 1 |  |  |  |
|  |  |  | *Bmal1^-/-^* | 23 | 1.261 | 0.1237 | *0.0405 |  |
| 3H | Unpaired *t*-test |  | *Bmal1^+/+^* | 25 | 1.1 |  |  |  |
|  |  |  | *Bmal1^-/-^* | 23 | 1.414 | 0.1113 | *0.0072 |  |
| 3I | Unpaired *t*-test |  | *Bmal1^+/+^* | 25 | 1.22 |  |  |  |
|  |  |  | *Bmal1^-/-^* | 23 | 1.5 | 0.1218 | *0.0339 |  |
| 3J | Unpaired *t*-test |  | *Bmal1^+/+^* | 25 | 4.34 |  |  |  |
|  |  |  | *Bmal1^-/-^* | 23 | 5.152 | 0.363 | *0.0234 |  |
| 4A | Two-way ANOVA |  | Untreated vs DSS *Bmal1^+/+^* |  |  |  |  |  |
|  |  |  | Untreated vs DSS *Bmal1^-/-^* | 3 (each) |  |  | *0.0399 | 5.245  (1, 11) |
|  |  | Tukey's test (*Bmal1^+/+^- Bmal1^-/-^*) | Untreated *Bmal1^+/+^ vs Bmal1^-/-^* |  |  |  | >0.9999 |  |
|  |  |  | Untreated vs DSS *Bmal1^+/+^* |  |  |  | *0.0147 |  |
|  |  |  | Untreated vs DSS *Bmal1^-/-^* |  |  |  | 0.9842 |  |
|  |  |  | DSS *Bmal1^+/+^ vs Bmal1^-/-^* |  |  |  | *0.0150 |  |
| 4B | Two-way ANOVA |  | Untreated vs DSS *Bmal1^+/+^* |  |  |  |  |  |
|  |  |  | Untreated vs DSS *Bmal1^-/-^* | 3 (each) |  |  | *0.0019 | 16.33  (1, 11) |
|  |  | Tukey's test (*Bmal1^+/+^- Bmal1^-/-^*) | Untreated *Bmal1^+/+^ vs Bmal1^-/-^* |  |  |  | >0.9999 |  |
|  |  |  | Untreated vs DSS *Bmal1^+/+^* |  |  |  | *0.0005 |  |
|  |  |  | Untreated vs DSS *Bmal1^-/-^* |  |  |  | 0.9993 |  |
|  |  |  | DSS *Bmal1^+/+^ vs Bmal1^-/-^* |  |  |  | *0.0003 |  |
| 4C | Two-way ANOVA |  | Untreated vs DSS *Bmal1^+/+^* |  |  |  |  |  |
|  |  |  | Untreated vs DSS *Bmal1^-/-^* | 3 (each) |  |  | 0.829 | 0.0492 (1, 10) |
|  |  | Tukey's test (*Bmal1^+/+^- Bmal1^-/-^*) | Untreated *Bmal1^+/+^ vs Bmal1^-/-^* |  |  |  | >0.9999 |  |
|  |  |  | Untreated vs DSS *Bmal1^+/+^* |  |  |  | *0.0085 |  |
|  |  |  | Untreated vs DSS *Bmal1^-/-^* |  |  |  | *0.0270 |  |
|  |  |  | DSS *Bmal1^+/+^ vs Bmal1^-/-^* |  |  |  | 0.987 |  |
| 4D | Two-way ANOVA |  | Untreated vs DSS *Bmal1^+/+^* |  |  |  |  |  |
|  |  |  | Untreated vs DSS *Bmal1^-/-^* | 3 (each) |  |  | 0.9997 | 1.05E-07  (1, 11) |
|  |  | Tukey's test (*Bmal1^+/+^- Bmal1^-/-^*) | Untreated *Bmal1^+/+^ vs Bmal1^-/-^* |  |  |  | 0.9171 |  |
|  |  |  | Untreated vs DSS *Bmal1^+/+^* |  |  |  | 0.9993 |  |
|  |  |  | Untreated vs DSS *Bmal1^-/-^* |  |  |  | 0.9994 |  |
|  |  |  | DSS *Bmal1^+/+^ vs Bmal1^-/-^* |  |  |  | 0.8631 |  |
| 4F | Unpaired *t*-test |  | *Bmal1^+/+^* | 5 | 0.0006 |  |  |  |
|  |  |  | *Bmal1^-/-^* | 4 | 0.0005 | 0.0002 | 0.8932 |  |
| 4G | Unpaired *t*-test |  | *Bmal1^+/+^* | 5 | 0.0272 |  |  |  |
|  |  |  | *Bmal1^-/-^* | 4 | 0.039 | 0.0108 | 0.3116 |  |
| 4H | Unpaired *t*-test |  | *Bmal1^+/+^* | 5 | 0.0537 |  |  |  |
|  |  |  | *Bmal1^-/-^* | 4 | 0.1022 | 0.0363 | 0.2236 |  |
| 4I | Unpaired *t*-test |  | *Bmal1^+/+^* | 5 | 1.171 |  |  |  |
|  |  |  | *Bmal1^-/-^* | 4 | 1.452 | 0.3348 | 0.4295 |  |
| 5A | Two-way ANOVA |  | ZT0 *Bmal1^+/+^* | 3 |  |  |  |  |
|  |  |  | ZT0 *Bmal1^-/-^* | 4 |  |  | *0.0042 | 5.004  (4, 25) |
|  |  | Tukey's test (*Bmal1^+/+^- Bmal1^-/-^*) | *Day 1* |  |  |  | >0.9999 |  |
|  |  |  | *Day 2* |  |  |  | 0.0795 |  |
|  |  |  | *Day 3* |  |  |  | 0.053 |  |
|  |  |  | *Day 4* |  |  |  | *0.0009 |  |
|  |  |  | *Day 5* |  |  |  | *0.0002 |  |
| 5B | Two-way ANOVA |  | ZT0 *Bmal1^+/+^* | 3 |  |  |  |  |
|  |  |  | ZT0 *Bmal1^-/-^* | 4 |  |  | 0.1199 | 2.036  (4, 25) |
|  |  | Tukey's test (*Bmal1^+/+^- Bmal1^-/-^*) | *Day 1* |  |  |  | >0.9999 |  |
|  |  |  | *Day 2* |  |  |  | 0.9311 |  |
|  |  |  | *Day 3* |  |  |  | 0.0838 |  |
|  |  |  | *Day 4* |  |  |  | 0.3186 |  |
|  |  |  | *Day 5* |  |  |  | *0.0496 |  |
| 5C | Two-way ANOVA |  | ZT0 *Bmal1^+/+^* | 3 |  |  |  |  |
|  |  |  | ZT0 *Bmal1^-/-^* | 4 |  |  | *0.0093 | 4.241  (4, 25) |
|  |  | Tukey's test (*Bmal1^+/+^- Bmal1^-/-^*) | *Day 1* |  |  |  | >0.9999 |  |
|  |  |  | *Day 2* |  |  |  | 0.3174 |  |
|  |  |  | *Day 3* |  |  |  | *0.0016 |  |
|  |  |  | *Day 4* |  |  |  | *0.0067 |  |
|  |  |  | *Day 5* |  |  |  | *0.0033 |  |
| 5D | Two-way ANOVA |  | ZT8 *Bmal1^+/+^* | 3 |  |  |  |  |
|  |  |  | ZT8 *Bmal1^-/-^* | 5 |  |  | 0.2763 | 1.345  (4, 30) |
|  |  | Tukey's test (*Bmal1^+/+^- Bmal1^-/-^*) | *Day 1* |  |  |  | >0.9999 |  |
|  |  |  | *Day 2* |  |  |  | 0.9163 |  |
|  |  |  | *Day 3* |  |  |  | 0.4185 |  |
|  |  |  | *Day 4* |  |  |  | 0.1072 |  |
|  |  |  | *Day 5* |  |  |  | 0.4965 |  |
| 5E | Two-way ANOVA |  | ZT8 *Bmal1^+/+^* | 3 |  |  |  |  |
|  |  |  | ZT8 *Bmal1^-/-^* | 5 |  |  | 0.2991 | 1.282  (4, 30) |
|  |  | Tukey's test (*Bmal1^+/+^- Bmal1^-/-^*) | *Day 1* |  |  |  | >0.9999 |  |
|  |  |  | *Day 2* |  |  |  | 0.318 |  |
|  |  |  | *Day 3* |  |  |  | 0.1581 |  |
|  |  |  | *Day 4* |  |  |  | 0.9446 |  |
|  |  |  | *Day 5* |  |  |  | 0.9446 |  |
| 5F | Two-way ANOVA |  | ZT8 *Bmal1^+/+^* | 3 |  |  |  |  |
|  |  |  | ZT8 *Bmal1^-/-^* | 5 |  |  | 0.164 | 1.755  (4, 30) |
|  |  | Tukey's test (*Bmal1^+/+^* -*Bmal1^-/‑^*) | *Day 1* |  |  |  | >0.9999 |  |
|  |  |  | *Day 2* |  |  |  | 0.9813 |  |
|  |  |  | *Day 3* |  |  |  | 0.0657 |  |
|  |  |  | *Day 4* |  |  |  | 0.5594 |  |
|  |  |  | *Day 5* |  |  |  | >0.9999 |  |
| 5G | Two-way ANOVA |  | ZT0 *Bmal1^+/+^* |  |  |  |  |  |
|  |  |  | ZT8 *Bmal1^+/+^* | 3 (each) |  |  | *0.0006 | 7.833  (4, 20) |
|  |  | Tukey's test (ZT0-ZT8) | *Day 1* |  |  |  | >0.9999 |  |
|  |  |  | *Day 2* |  |  |  | 0.9445 |  |
|  |  |  | *Day 3* |  |  |  | >0.9999 |  |
|  |  |  | *Day 4* |  |  |  | *0.0252 |  |
|  |  |  | *Day 5* |  |  |  | *<0.0001 |  |
| 5H | Two-way ANOVA |  | ZT0 *Bmal1^+/+^* |  |  |  |  |  |
|  |  |  | ZT8 *Bmal1^+/+^* | 3 (each) |  |  | 0.4930 | 0.8810 (4, 20) |
|  |  | Tukey's test (ZT0-ZT8) | *Day 1* |  |  |  | >0.9999 |  |
|  |  |  | *Day 2* |  |  |  | >0.9999 |  |
|  |  |  | *Day 3* |  |  |  | 0.9906 |  |
|  |  |  | *Day 4* |  |  |  | >0.9999 |  |
|  |  |  | *Day 5* |  |  |  | 0.6379 |  |
| 5I | Two-way ANOVA |  | ZT0 *Bmal1^+/+^* | 3 |  |  |  |  |
|  |  |  | ZT8 *Bmal1^+/+^* | 3 |  |  | 0.1610 | 1.839  (4, 20) |
|  |  | Tukey's test (ZT0-ZT8) | *Day 1* |  |  |  | >0.9999 |  |
|  |  |  | *Day 2* |  |  |  | 0.9743 |  |
|  |  |  | *Day 3* |  |  |  | 0.9743 |  |
|  |  |  | *Day 4* |  |  |  | 0.7813 |  |
|  |  |  | *Day 5* |  |  |  | *0.0367 |  |
| 6A | Unpaired *t*-test |  | *ZT4 Bmal1^+/+^* | 5 | 7.33E-03 |  |  |  |
|  |  |  | *ZT4 Bmal1^-/-^* | 4 | 4.35E-03 | 4.21E-03 | 0.5191 |  |
|  | Unpaired *t*-test |  | *ZT16 Bmal1^+/+^* | 3 | 0.0142 |  |  |  |
|  |  |  | *ZT16 Bmal1^-/-^* | 3 | 0.01214 | 0.003118 | 0.4409 |  |
| 6B | Unpaired *t*-test |  | *ZT4 Bmal1^+/+^* | 5 | 2.06E-07 |  |  |  |
|  |  |  | *ZT4 Bmal1^-/-^* | 3 | 2.78E-07 | 1.84E-07 | 0.7145 |  |
|  | Unpaired *t*-test |  | *ZT16 Bmal1^+/+^* | 3 | 0.006333 |  |  |  |
|  |  |  | *ZT16 Bmal1^-/-^* | 3 | 0.002275 | 0.092 | 0.2605 |  |
| 6C | Unpaired *t*-test |  | *ZT4 Bmal1^+/+^* | 5 | 8.00E-09 |  |  |  |
|  |  |  | *ZT4 Bmal1^-/-^* | 4 | 3.17E-08 | 5.15E-09 | *0.0100 |  |
|  | Unpaired *t*-test |  | *ZT16 Bmal1^+/+^* | 3 | 0.0028 |  |  |  |
|  |  |  | *ZT16 Bmal1^-/-^* | 3 | 0.0043 | 0.002145 | 0.2719 |  |
| 6D | Unpaired *t*-test |  | *Bmal1^+/+^* | 3 | 1.628 |  |  |  |
|  |  |  | *Bmal1^-/-^* | 3 | 5.583 | 0.894 | *0.0115 |  |
| 6E | One-way ANOVA |  | Bmal1+/+ |  |  |  | 0.2388 | 1.493 |
|  | One-way ANOVA |  | Bmal1-/- |  |  |  | 0.1274 | 2.039 |
|  | Two-way ANOVA |  | ZT0, 4, 8, 12, 16, 20 *Bmal1^+/+^* | ZT0, 8=5 |  |  | 0.5024 | 0.8830 (5, 36) |
|  |  |  |  | ZT4 =6 |  |  |  |  |
|  |  |  |  | Rest=3 |  |  |  |  |
|  |  |  | ZT0, 4, 8, 12, 16, 20 *Bmal1^-/-^* | ZT0, 20=5 |  |  |  |  |
|  |  |  |  | ZT12=4 |  |  |  |  |
|  |  |  |  | Rest=3 |  |  |  |  |
|  |  | Tukey's test (*Bmal1^+/+^* -*Bmal1^-/‑^*) | ZT0 |  |  |  | >0.9999 |  |
|  |  |  | ZT4 |  |  |  | 0.6283 |  |
|  |  |  | ZT8 |  |  |  | 0.777 |  |
|  |  |  | ZT12 |  |  |  | >0.9999 |  |
|  |  |  | ZT16 |  |  |  | >0.9999 |  |
|  |  |  | ZT20 |  |  |  | 0.6339 |  |
| 6G | One-way ANOVA |  | Bmal1+/+ |  |  |  | 0.9109 | 0.0948 |
|  | One-way ANOVA |  | Bmal1-/- |  |  |  | 0.2548 | 1.732 |
|  | Two-way ANOVA |  | ZT4, 12, 20 *Bmal1^+/+^* |  |  |  | 0.4025 | 0.9027 (2, 12) |
|  |  |  | ZT4, 12, 20 *Bmal1^-/-^* | 3 (each) |  |  |  |  |
|  |  | Tukey's test (*Bmal1^+/+^* -*Bmal1^-/‑^*) | ZT4 |  |  |  | 0.2262 |  |
|  |  |  | ZT12 |  |  |  | 0.9971 |  |
|  |  |  | ZT20 |  |  |  | 0.8339 |  |
| 6H | Unpaired *t*-test |  | *Bmal1^+/+^* |  | 2224 |  |  |  |
|  |  |  | *Bmal1^-/-^* | 9 (each) | 2773 | 230.9 | *0.0360 |  |
| 6I | One-way ANOVA |  | ZT4, 12, 20 *Bmal1^+/+^* | 3 (each) |  |  | *0.0474 | 5.287 |
|  | Two-way ANOVA |  | ZT4, 12, 20 *Bmal1^+/+^* |  |  |  |  |  |
|  |  |  | ZT4, 12, 20 *Bmal1^-/-^* | 3 (each) |  |  | *0.0053 | 8.362 (2, 12) |
|  |  | Tukey's test (*Bmal1^+/+^* -*Bmal1^-/‑^*) | ZT4 |  |  |  | 0.9794 |  |
|  |  |  | ZT12 |  |  |  | *0.0056 |  |
|  |  |  | ZT20 |  |  |  | >0.9999 |  |
| 7B | Two-way ANOVA |  | ZT0, 12 *Bmal1^+/+^* |  |  |  |  |  |
|  |  |  | ZT0, 12 *Bmal1^-/-^* | 3 (each) |  |  | *0.0096 | 11.43 (1, 8) |
|  |  | Tukey's test | ZT0 *Bmal1^+/+^* -*Bmal1^-/‑^* |  |  |  | *0.0031 |  |
|  |  |  | ZT0, 12 *Bmal1^+/+^* |  |  |  | *0.0253 |  |
| 7D | One-way ANOVA |  | ZT0, 4, 8, 12, 16, 20 *Bmal1^+/+^* | 3 (each) |  |  | *0.0022 | 7.416 |
|  | Two-way ANOVA |  | ZT0, 4, 8, 12, 16, 20 *Bmal1^+/+^* |  |  |  |  |  |
|  |  |  | ZT0, 4, 8, 12, 16, 20  *Bmal1^-/-^* | 3 (each) |  |  | *0.0021 | 5.264 (5, 24) |
|  |  | Tukey's test | ZT0 |  |  |  | *0.0003 |  |
|  |  | (*Bmal1^+/+^* -*Bmal1^-/‑^*) |  |  |  |  |  |  |
|  |  |  | ZT4 |  |  |  | 0.0521 |  |
|  |  |  | ZT8 |  |  |  | 0.9267 |  |
|  |  |  | ZT12 |  |  |  | >0.9999 |  |
|  |  |  | ZT16 |  |  |  | >0.9999 |  |
|  |  |  | ZT20 |  |  |  | 0.9877 |  |
|  | Chronos-Fit |  | ZT0, 4, 8, 12, 16, 20 *Bmal1^+/+^* |  |  |  | *0.0011 | 11.1 |
|  |  |  | ZT0, 4, 8, 12, 16, 20  *Bmal1^-/-^* |  |  |  | No fit |  |
| 7F | One-way ANOVA |  | ZT0, 4, 8, 12, 16, 20 *Bmal1^+/+^* | 3 (each) |  |  | 0.2911 | 1.403 |
|  | Two-way ANOVA |  | ZT0, 4, 8, 12, 16, 20 *Bmal1^+/+^* |  |  |  |  |  |
|  |  |  | ZT0, 4, 8, 12, 16, 20 *Bmal1^-/-^* | 3 (each) |  |  | 0.5329 | 0.8444 (5, 22) |
| 8C | Unpaired *t*-test |  | *Bmal1^+/+^* |  | 73.24 |  |  |  |
|  |  |  | *Bmal1^-/-^* | 3 (each) | 20.38 | 11.18 | *0.0091 |  |
| 8D | Unpaired *t*-test |  | *Bmal1^+/+^* |  | 23.33 |  |  |  |
|  |  |  | *Bmal1^-/-^* | 3 (each) | 8.81 | 2.75 | *0.0062 |  |
| 8F | Unpaired *t*-test |  | *Bmal1^+/+^* |  | 58.14 |  |  |  |
|  |  |  | *Bmal1^-/-^* | 3 (each) | 19.05 | 13.99 | *0.0491 | ­ |
| 8G | Unpaired *t*-test |  | *Bmal1^+/+^* |  | 29.9 |  |  |  |
|  |  |  | *Bmal1^-/-^* | 3 (each) | 7.81 | 6.71 | *0.0301 |  |
| 8I | Unpaired *t*-test |  | *Bmal1^+/+^* |  | 13.42 |  |  |  |
|  |  |  | *Bmal1^-/-^* | 3 (each) | 7.6 | 2.079 | *0.0489 |  |
| 8J | Unpaired *t*-test |  | *Bmal1^+/+^* |  | 10.43 |  |  |  |
|  |  |  | *Bmal1^-/-^* | 3 (each) | 6.567 | 2.889 | 0.2521 |  |
| 8L | Unpaired *t*-test |  | *Bmal1^+/+^* |  | 32.38 |  |  |  |
|  |  |  | *Bmal1^-/-^* | 3 (each) | 26.29 | 11.45 | 0.6226 |  |
| 8M | Unpaired *t*-test |  | *Bmal1^+/+^* |  | 30.19 |  |  |  |
|  |  |  | *Bmal1^-/-^* | 3 (each) | 15 | 5.346 | *0.0468 |  |
| 9B | Unpaired *t*-test |  | *Bmal1^+/+^* |  | 314.6 |  |  |  |
|  |  |  | *Bmal1^-/-^* | 3 (each) | 6993 | 1407 | *0.0090 |  |
| 9C | Unpaired *t*-test |  | *Bmal1^+/+^* |  | 24.07 |  |  |  |
|  |  |  | *Bmal1^-/-^* | 3 (each) | 15.32 | 2.772 | *0.0344 |  |
| 9D | Unpaired *t*-test |  | *Bmal1^+/+^* |  | 120.4 |  |  |  |
|  |  |  | *Bmal1^-/-^* | 3 (each) | 184.3 | 10.06 | *0.0032 |  |
| 9E | Unpaired *t*-test |  | *Bmal1^+/+^* |  | 75.42 |  |  |  |
|  |  |  | *Bmal1^-/-^* | 3 (each) | 165.7 | 28.9 | *0.0354 |  |
| Sup. 1A | Unpaired *t*-test |  | *Bmal1^+/+^* | 25 | 0.48 |  |  |  |
|  |  |  | *Bmal1^-/-^* | 23 | 0.5 | 0.02087 | 0.3429 |  |
| Sup. 1B | Unpaired *t*-test |  | *Bmal1^+/+^* | 25 | 0.48 |  |  |  |
|  |  |  | *Bmal1^-/-^* | 23 | 0.4783 | 0.05181 | 0.9734 |  |
| Sup. 2A | Two-way ANOVA |  | ZT0 *Bmal1^+/+^* | 3 |  |  |  |  |
|  |  |  | ZT0 *Bmal1^-/-^* | 4 |  |  | 0.2789 | 1.351 (4, 25) |
|  |  | Tukey's test (*Bmal1^+/+^- Bmal1^-/-^*) | *Day 1* |  |  |  | >0.9999 |  |
|  |  |  | *Day 2* |  |  |  | 0.9997 |  |
|  |  |  | *Day 3* |  |  |  | 0.1912 |  |
|  |  |  | *Day 4* |  |  |  | 0.9891 |  |
|  |  |  | *Day 5* |  |  |  | >0.9999 |  |
| Sup. 2B | Two-way ANOVA |  | ZT8 *Bmal1^+/+^* | 3 |  |  |  |  |
|  |  |  | ZT8 *Bmal1^-/-^* | 5 |  |  | 0.0577 | 2.575 (4, 30) |
|  |  | Tukey's test (*Bmal1^+/+^- Bmal1^-/-^*) | *Day 1* |  |  |  | >0.9999 |  |
|  |  |  | *Day 2* |  |  |  | 0.99 |  |
|  |  |  | *Day 3* |  |  |  | 0.7591 |  |
|  |  |  | *Day 4* |  |  |  | >0.9999 |  |
|  |  |  | *Day 5* |  |  |  | 0.3082 |  |
